# COVID-19 among people experiencing homelessness in England: a modelling study

**DOI:** 10.1016/S2213-2600(20)30396-9

**Published:** 2020-12

**Authors:** Dan Lewer, Isobel Braithwaite, Miriam Bullock, Max T Eyre, Peter J White, Robert W Aldridge, Alistair Story, Andrew C Hayward

**Affiliations:** a UCL Collaborative Centre for Inclusion Health, University College London, London, UK; b UCL Public Health Data Science Research Group, Institute of Health Informatics, University College London, London, UK; c Centre for Health Informatics, Computing, and Statistics, Lancaster University Medical School, Lancaster, UK; d MRC Centre for Global Infectious Disease Analysis, Department of Infectious Disease Epidemiology, Imperial College London, London, UK; e Modelling and Economics Unit, National Infection Service, Public Health England, London, UK; f Find and Treat, University College London Hospitals NHS Foundation Trust, London, UK

## Abstract

**Background:**

People experiencing homelessness are vulnerable to COVID-19 due to the risk of transmission in shared accommodation and the high prevalence of comorbidities. In England, as in some other countries, preventive policies have been implemented to protect this population. We aimed to estimate the avoided deaths and health-care use among people experiencing homelessness during the so-called first wave of COVID-19 in England—ie, the peak of infections occurring between February and May, 2020—and the potential impact of COVID-19 on this population in the future.

**Methods:**

We used a discrete-time Markov chain model of severe acute respiratory syndrome coronavirus 2 (SARS-CoV-2) infection that included compartments for susceptible, exposed, infectious, and removed individuals, to explore the impact of the pandemic on 46 565 individuals experiencing homelessness: 35 817 living in 1065 hostels for homeless people, 3616 sleeping in 143 night shelters, and 7132 sleeping outside. We ran the model under scenarios varying the incidence of infection in the general population and the availability of prevention measures: specialist hotel accommodation, infection control in homeless settings, and mixing with the general population. We divided our scenarios into first wave scenarios (covering Feb 1–May 31, 2020) and future scenarios (covering June 1, 2020–Jan 31, 2021). For each scenario, we ran the model 200 times and reported the median and 95% prediction interval (2·5% and 97·5% quantiles) of the total number of cases, the number of deaths, the number hospital admissions, and the number of intensive care unit (ICU) admissions.

**Findings:**

Up to May 31, 2020, we calibrated the model to 4% of the homeless population acquiring SARS-CoV-2, and estimated that 24 deaths (95% prediction interval 16–34) occurred. In this first wave of SARS-CoV-2 infections in England, we estimated that the preventive measures imposed might have avoided 21 092 infections (19 777–22 147), 266 deaths (226–301), 1164 hospital admissions (1079–1254), and 338 ICU admissions (305–374) among the homeless population. If preventive measures are continued, we projected a small number of additional cases between June 1, 2020, and Jan 31, 2021, with 1754 infections (1543–1960), 31 deaths (21–45), 122 hospital admissions (100–148), and 35 ICU admissions (23–47) with a second wave in the general population. However, if preventive measures are lifted, outbreaks in homeless settings might lead to larger numbers of infections and deaths, even with low incidence in the general population. In a scenario with no second wave and relaxed measures in homeless settings in England, we projected 12 151 infections (10 718–13 349), 184 deaths (151–217), 733 hospital admissions (635–822), and 213 ICU admissions (178–251) between June 1, 2020, and Jan 31, 2021.

**Interpretation:**

Outbreaks of SARS-CoV-2 in homeless settings can lead to a high attack rate among people experiencing homelessness, even if incidence remains low in the general population. Avoidance of deaths depends on prevention of transmission within settings such as hostels and night shelters.

**Funding:**

National Institute for Health Research, Wellcome, and Medical Research Council.

## Introduction

The current pandemic of the viral respiratory disease COVID-19 does not pose equal risks to all parts of society. People experiencing homelessness might be vulnerable to infection due to transmission risks in homeless accommodation settings and barriers to preventive behaviours such as regular handwashing and avoiding contact with others. Outbreaks of COVID-19 have been documented in shelters in the USA.^1^, ^2^ Similarly, during historic pandemic influenza seasons, particularly large spikes in hospitalisations have been observed in homeless populations.^3^ In addition to risks related to infection, people experiencing homelessness might be at increased risk of severe COVID-19 due to high prevalence of long-term health conditions.^4^, ^5^ Cohort studies of people experiencing homelessness done before the pandemic show large numbers of excess deaths due to cardiovascular and chronic respiratory diseases,^6^, ^7^ which are diseases that increase the risk of severe COVID-19.^8^, ^9^

England experienced a so-called first wave of COVID-19 in the general population in early 2020. The first case was reported on Jan 29, cases peaked in the first week of April, and incidence had returned a low level by the end of May, 2020.^10^ During this first wave, an estimated 5–6% of the general population of England developed antibodies to severe acute respiratory syndrome coronavirus 2 (SARS-CoV-2).^11^, ^12^ At the time of publication, incidence of COVID-19 in the general population remains much lower than during this first wave, but there is emerging evidence of another increase in cases.^13^

**Research in context**

**Evidence before this study**

Homeless populations have poor health outcomes, with epidemiological studies showing high prevalence of cardiovascular diseases, respiratory diseases, and chronic infections, and all-cause mortality rates of three to six times those seen in the general population. There is evidence that homeless populations have particularly large spikes in hospitalisations during pandemic influenza seasons, suggesting vulnerability to viral respiratory infections. The risk of COVID-19 outbreaks in homeless accommodation settings has prompted some interventions to reduce transmission risks, but the impact to date, and potential future impact, are unclear. We searched PubMed, pre-print servers (*medRxiv, bioRxiv*, and *arXiv*) and Google Scholar from Dec 1, 2019, to Aug 3, 2020, using search terms related to homelessness (“homeless*”, “rough sleep*”, “street sleep*”, [“inadequate*”, “insecure*”, “precarious*”, “unstabl*”] and “hous*”, “houseless”, “roofless”), COVID-19 (“COVID*”, “SARS-CoV-2”, “novel coronavirus”, “nCoV-2019”, “respiratory”), and outbreaks (“outbreak*, “disease control”, “health protection”). We had no restrictions on study type. We identified 14 studies. Five studies documented outbreaks of COVID-19 in homeless shelters (four in the USA and one in France), with control measures supported by public health authorities. A study including four shelters in France found that infection was associated with residing in the shelter with the most beds per room. One report from a shelter in Canada showed that preventive measures including isolation of symptomatic residents appeared to prevent secondary cases. A study in Boston, USA, showed that the incidence of COVID-19 among homeless people was substantially higher than in the general population. Although some data from homeless settings have been collected, mostly in North America, and several policy reviews have described measures taken in respect of homeless populations, the impact of COVID-19 on people experiencing homelessness remains largely undocumented.

**Added value of this study**

We used a stochastic epidemic model to explore the potential incidence of COVID-19 among people experiencing homeless in England, as well as the number of deaths and the need for hospital and intensive care beds. Evidence from organisations working with homeless people in England suggests that there has been a low number of deaths of homeless people from COVID-19 to date, which we attributed to reduced mixing with the general population during the lockdown intervention, increased infection control in hostels, and accommodation of people sleeping rough in commercial hotels. Together, we find that these interventions might have reduced deaths in the homeless population by 92% during the first wave of COVID-19. We show that the number of additional deaths is likely to be kept low if these measures are continued, but lifting of these measures could lead to outbreaks and larger numbers of deaths regardless of incidence in the general population.

**Implications of all the available evidence**

The risk of outbreaks of infectious disease in homeless settings is well known. COVID-19 outbreaks remain likely in these settings, even when incidence is low in the general population. Outbreaks can be prevented by providing stable single-room accommodation and by heightening infection control measures in homeless settings. These interventions can avoid large numbers of deaths.

There are few data on the impact of the disease on people experiencing homelessness. Some data are available from surveillance of homeless accommodation projects in London (appendix p 5). This includes testing of people in homeless settings in London, which suggests that incidence of COVID-19 followed the general population trend, peaking in early April. Sites participating in a surveillance programme, with a total of 6075 residents, reported four deaths due to COVID-19 between March 1 and June 16, 2020 (unpublished). The infection–fatality ratio (IFR) is likely to be higher in people experiencing homelessness than in the general population due to health-related vulnerabilities such as chronic respiratory problems.^4^, ^5^ Assuming an IFR of 1·6%, which accounts for this increased vulnerability, the surveillance data imply a cumulative incidence of 4% (95% CI 1–10; see appendix p 7 for details of this estimate). These data suggest that the impact of COVID-19 on people experiencing homelessness has been less than expected, although there is high uncertainty around these estimates and they do not include those who remained sleeping rough during the first wave.

We identified three potential preventive factors in the homeless population: (1) a programme of residential interventions that might have reduced transmission among homeless people (panel), (2) reduced mixing with the general population due to restrictions on movement and activities (ie, lockdown), and (3) infection control measures in hostels and other homeless settings, such as closing of communal areas, promotion of hand hygiene, and advising residents and staff to limit contact with others (ie, physical distancing).

Panel **Support for homeless people during COVID-19: COVID-PROTECT and COVID-CARE**

Some countries have offered additional support to homeless people during the pandemic. Health and housing authorities in England developed a plan with two main elements: (1) provision of single room own-bathroom accommodation to homeless adults (called COVID-PROTECT); and (2) testing and medically supported accommodation for those with symptoms (called COVID-CARE).^14^ Similar models have also been implemented in other places; for example, New York City and Los Angeles County have used hotel rooms to shelter vulnerable homeless people and established separate medical sheltering facilities for those with symptoms.^15^, ^16^

On March 26, 2020, the UK Government instructed local authorities to provide accommodation for people sleeping rough during the pandemic.^17^ Dormitory-style night shelters were subsequently closed and COVID-PROTECT accommodation focused on people sleeping in these facilities and those sleeping rough. COVID-PROTECT accommodation has mainly been in commercial hotels that have been left otherwise vacant during the pandemic. Most people who were living in homeless hostels before the pandemic have stayed in their existing location, with increased infection control. Approaches to COVID-CARE have differed. In some areas, people have been transferred to COVID-CARE on the basis of symptoms and then discharged after two negative tests, and in others, people have been transferred after receiving a positive result. At the time of publication, COVID-CARE facilities have closed due to small numbers of cases, whereas many COVID-PROTECT facilities remain open. On June 3, 2020, the UK Government reported that 14 610 individuals had been accommodated by that point,^18^ including people sleeping outside, sleeping in night shelters, or at risk of sleeping rough. Further funding for local authorities to provide longer-term accommodation was announced on June 24.^19^

We aimed to model the number of infections, deaths, and hospital admissions that have been avoided as a result of these preventive factors, and to estimate the potential impact of COVID-19 on this population under different future scenarios.

## Methods

### Transition model

**Figure 1:**
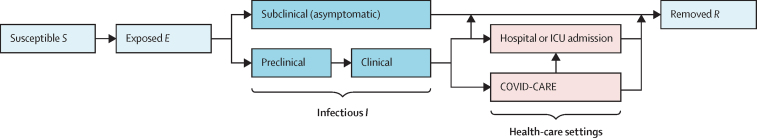
State transitions in the model Individuals are classified into susceptible, exposed, infectious (asymptomatic or symptomatic), and removed states (ie, no longer susceptible). Light and dark blue boxes represent individuals in community settings: living in a hostel, sleeping in a night shelter, sleeping outside, or staying in a COVID-PROTECT hotel. Pink boxes represent individuals in health-care settings (COVID-CARE or hospital). Each of these locations is modelled as a number of separate closed subgroups, based on data about homeless accommodation in England. ICU=intensive care unit.

We developed a discrete-time Markov chain^20^ model to simulate stochastic daily transitions of individuals between four compartments—susceptible to COVID-19, exposed, infectious, or recovered (including immune or died)—following the SEIR (susceptible-exposed-infectious-removed) approach^21^ to modelling of infectious diseases. We also modelled the location of individuals as being in a community setting including hostels, night shelters, and sleeping rough (ie, sleeping outside); in COVID-PROTECT or COVID-CARE (panel); or in hospital. State transitions are summarised in figure 1, with a more detailed flow-chart and key assumptions provided in the appendix (pp 13, 16–17). Ethics review and approval was not required for this study because it only used data that are publicly available or have been anonymised and aggregated.

### The homeless population

The size and structure of the homeless population is difficult to estimate, not least because there are different types of homelessness.^22^ We considered three groups: people living in hostels for single homeless people (typically single bedroom accommodation), people sleeping rough, and people sleeping in night shelters (multiple beds in one large room). A census of providers of accommodation for single homeless people suggested there were 35 817 people living in 1065 hostels in England in 2019, and the median hostel size was 21 beds (IQR 12–38).^23^ There are few estimates available of the number of people sleeping rough; we used an official government count^24^ in combination with an estimate of the number of people not captured by this count, based on data from a multi-agency database, to produce an estimate of 10 748 people currently sleeping rough in England. We assumed that people sleeping in night shelters are a subset of this population. We used data on the number and size of night shelters in London to estimate that 3616 of 10 748 people sleeping rough in England are sleeping in 143 night shelters, and these night shelters have a median of 15 beds (IQR 14–25) each. Our total population was therefore 46 565 people. Further detail on these estimates is provided in the appendix (p 2).

Most studies of single people experiencing homelessness suggest that the majority are men and the average age is around 40–50 years.^5^, ^25^ To inform assumptions about risk of severe COVID-19, we used the age and sex profile of clients of a homeless charity in England (St Mungo's). 77% of this population were male and the majority were aged 30–49 years (appendix p 7).

### SARS-CoV-2 infection

We assumed that people experiencing homelessness could acquire SARS-CoV-2 either from mixing with the general population or transmission within homeless settings following introduction of SARS-CoV-2 from mixing with the general population.

We modelled the risk associated with mixing with the general population as the incidence of infection in the general population multiplied by a fixed parameter *m*, which we assumed to be less than 1 owing to lockdown measures implemented during the first wave, which was modelled as Feb 1 to May 31, 2020. For example, *m*=0·5 would lead to 5·4% × 0·5=2·7% of the homeless population being infected due to mixing with the general population alone, where 5·4% is the estimated cumulative incidence in the general population after the first wave. We estimated the daily incidence in the general population using prevalence of antibodies at the end of the first wave (appendix p 9).^11^

Cases arising due to mixing with the general population acted as seeds for transmission within homeless settings, which we modelled as closed groups. We assumed that transmission in the community occurs within 1569 subgroups, consisting of the 1065 hostels and 143 night shelters described above, and subgroups of people sleeping rough. We did not have evidence upon which to base the structure of mixing among people sleeping rough, and therefore created synthetic subgroups of sizes one to 100, where the group size was sampled with probabilities of the inverse of the size, meaning there were more small groups than large groups. This generated 361 subgroups of median size nine (IQR 2–28). We assumed homogeneous mixing within subgroups, but no mixing or transmission between them.

For each day, we calculated the so-called force of infection (ie, the risk of infection per susceptible individual) within each subgroup, based on the infectivity of each infectious individual, the duration of infectiousness for those individuals, and the number of individuals that were infectious in that subgroup. Infectivity, expressed as the expected number of secondary cases from an infectious individual if all contacts were susceptible, varied across infectious individuals, and was sampled from a negative binomial distribution with a fixed mean (reproduction number *R*_0_) and a dispersion parameter *k*. For individuals in hostels, we set *R*_0_ to 2·5 in scenarios with no preventive measures, guided by published estimates of *R*_0_ for SARS-CoV-2 in the general population and in closed settings.^26^, ^27^ We assumed that night shelters would be associated with greater risk and set *R*_0_ to 1·5 times this value (ie, *R*_0_=3·75), whereas sleeping rough would be associated with lower risk due to being outdoors, for which we set *R*_0_ to 0·75 times the hostel value (ie, *R*_0_=1·88). For our base scenarios, we modelled transmission as being less dispersed than in the general population^28^ (although still with a high degree of dispersion) because homeless people are a subpopulation with shared characteristics and are likely to have less variation in contact rates. This approach allows for variation in the likelihood and size of outbreaks, and for so-called super-spreader events (ie, where one individual infects many others). In addition to these variables, the number of transmission events is subject to stochastic variation.

### Course of disease

We used assumptions regarding the duration of exposed and infectious statuses and hospital admission from an existing model of COVID-19 in the general population of the UK.^26^ We classified cases as asymptomatic, mild (requiring basic clinical observation and self-isolation), moderate (requiring hospital admission), and severe (requiring intensive care unit [ICU] admission), with higher mortality risks for more severe cases. We assumed an overall IFR of 1·62%. This value was based on existing estimates of the IFR for patients of a similar age and sex in the general population, with adjustment to account for the health-related vulnerability of the homeless population. The adjustment was based on published evidence of the increased background mortality risk among homeless people,^29^, ^30^ as overall mortality risk may represent vulnerability to COVID-19 (appendix p 7). This approach accounts for increased risks due to comorbidities, whether diagnosed or not, as well as accounting for risk factors such as frailty and poor nutrition. Our assumptions regarding disease severity are shown in table 1. Individuals with different disease severities, including asymptomatic cases, were assumed to have equal infectiousness.

**Table 1:** Assumptions regarding severity, infection–fatality ratio, and health-care use in people experiencing homelessness

	**Proportion of cases**	**Infection–fatality ratio**	**Health-care use**
Asymptomatic	40·0%	0·00%	None
Mild	53·8%	0·28%	COVID-CARE (if the patient accepts)
Moderate	4·4%	15·00%	Hospital admission
Severe	1·8%	45·00%	Intensive care unit
Total	100·0%	1·62%	..

### Intervention

We assumed that COVID-PROTECT and COVID-CARE opened on March 1, 2020 (panel), and that people sleeping outside or sleeping in night shelters are offered COVID-PROTECT on a random day between March 1 and March 29, if they are susceptible or asymptomatic on that day. We assumed that all individuals sleeping in night shelters accept COVID-PROTECT, while individuals sleeping outside have an 80% chance of accepting. We modelled COVID-PROTECT as 106 hotels of sizes 22–200 beds, with individuals moved to a hotel at random. Individuals remain in COVID-PROTECT until the intervention closes (table 2) or self-discharge. Self-discharge was modelled as a daily risk of 0·6%, with individuals returning to their original community subgroup. Asymptomatic or pre-symptomatic individuals might be accommodated in COVID-PROTECT, leading to infectious individuals in this setting. We assumed that transmission could occur in COVID-PROTECT (with *R*_0_ set at 0·75) within each separate hotel, in the same way as for community settings. Individuals who develop symptomatic COVID-19 in community settings or in a COVID-PROTECT hotel are offered COVID-CARE on the day after developing symptoms, and we assumed they have an 80% chance of accepting this. In our model, no transmission occurs in COVID-CARE, because we assumed that all residents are already infected at the time of admission. At the end of symptoms, individuals are discharged from COVID-CARE to their community subgroup, or offered COVID-PROTECT if they are eligible. A detailed flowchart is provided in the appendix, along with a table of key assumptions (pp 13, 16–17).

**Table 2:** Scenario parameters

	**Dates**	**R_0_ in community homeless settings**	**Mixing parameter *m***	**COVID-CARE and COVID-PROTECT**	**General population cumulative incidence**
**First wave scenarios**					
Scenario A: first wave; base scenario (preventive measures in place)	Feb 1–May 31, 2020	0·75	0·5	Open from March 1, 2020	5·4%
Scenario B: first wave; do nothing (counterfactual)	Feb 1–May 31, 2020	2·5 in hostels, 1·88 for rough sleepers, 3·75 in night shelters	1·0	Closed	5·4%
**No second wave scenarios**					
Scenario C: no second wave; retain measures	June 1, 2020–Jan 31, 2021	0·75	0·5	Open throughout	Low ongoing transmission
Scenario D: no second wave; lift measures	June 1, 2020–Jan 31, 2021	2·5 in hostels, 1·88 for rough sleepers, 3·75 in night shelters	1·0	Closed from Aug 1, 2020	Low ongoing transmission
Scenario E: no second wave; lift measures except for COVID-CARE and COVID-PROTECT	June 1, 2020–Jan 31, 2021	2·5 in hostels, 1·88 for rough sleepers, 3·75 in night shelters	1·0	Open throughout	Low ongoing transmission
**Second wave scenarios**					
Scenario F: sharp second wave; retain measures	June 1, 2020–Jan 31, 2021	0·75	0·5	Open throughout	Additional 2·7%
Scenario G: sharp second wave; lift measures, reduced mixing with general population	June 1, 2020–Jan 31, 2021	2·5 in hostels, 1·88 for rough sleepers, 3·75 in night shelters	0·5	Closed from Aug 1, 2020	Additional 2·7%
Scenario H: flatter second wave; lift measures, reduced mixing with general population	June 1, 2020–Jan 31, 2021	2·5 in hostels, 1·88 for rough sleepers, 3·75 in night shelters	0·5	Closed from Aug 1, 2020	Additional 2·7%

The general population cumulative incidence is used to estimate the daily incidence of SARS-CoV-2, which informs the chance of homeless settings being seeded with index cases. For low ongoing transmission, we assumed 5000 cases per day in England. SARS-CoV-2=severe acute respiratory syndrome coronavirus 2.

### Scenarios

We divided our scenarios into first wave scenarios (covering Feb 1–May 31, 2020) and future scenarios (covering June 1, 2020–Jan 31, 2021). We defined two first wave scenarios: A and B (table 2). Scenario A is a historical estimate of the pandemic in the homeless population, with preventive measures implemented. Our best estimate of the cumulative incidence of SARS-CoV-2 among homeless people in England is 4% on May 31, 2020 (appendix p 5). We set *R*_0_ to 0·75 for this period and then determined the value of *m* that would produce a cumulative incidence of 4%. Scenario B is a counterfactual scenario with general population lockdown measures in place but no additional public health interventions for the homeless population; for this scenario, *m* was set to 1 and *R*_0_ values were not reduced.

We ran the model under six future scenarios (table 2). In scenarios C, D, and E, there is no second wave in the general population: the incidence in the general population remains low (ie, 5000 cases per day), which informs the risk of a homeless settings being seeded with a new case. Scenario C involves continuation of all prevention measures, scenario D involves lifting of all prevention measures, and scenario E involves lifting of prevention measures except for COVID-CARE and COVID-PROTECT. In scenarios F, G, and H, there is a second wave in the general population, each with half the total number of infections as the first wave and maximum incidence on Nov 1, 2020 (appendix p 9). In scenarios F and G, there is a sharp second wave, with the same duration as the first wave: scenario F involves continuation of all prevention measures, and scenario G involves lifting of prevention measures. In scenario G, *m* is kept at 0·5 because restrictions on movement and activities in the general population are likely if community transmission increases substantially. Scenario H is the same as scenario G, but the second wave has a longer and flatter profile, with three times the duration of the first wave. All future scenarios continue from scenario A, with infections, deaths and health-care use reported from June 1, 2020 (rather than for the full model duration including scenario A).

For each scenario, we ran the model 200 times and reported the median and 95% prediction interval (2·5% and 97·5% quantiles) of the total number of cases, the number of deaths, the number hospital admissions, the number of ICU admissions, and the cumulative incidence of infections. To check the stability of results, we compared distribution of results in the first and second half of the model runs in each scenario. Median values and distributions across halves were very similar.

### Sensitivity analyses

We did univariable sensitivity analyses in which model parameters were substituted with low and high values in a specified base scenario. For these analyses, we varied the IFR, *R*_0_ in community settings, *R*_0_ in COVID-PROTECT, the dispersion parameter *k*, the size of the first wave in the general population, the proportion that accept COVID-PROTECT or COVID-CARE when offered, the risk of self-discharge from COVID-PROTECT and COVID-CARE, and the degree of mixing with the general population when there are restrictions on movement (*m*). We followed this with a multivariable probabilistic sensitivity analysis in which we included the three parameters that had the greatest influence on the numbers of infections and deaths in univariable analysis, as well as the mixing variable *m*, and varied these simultaneously (appendix pp 11–12).

We built the model in R (version 4.0.0). Code is available online.

### Role of the funding source

The funders of the study had no role in study design, data collection, data analysis, data interpretation, writing of the report, or the decision to submit the paper for publication. All authors had full access to all the data in the study and had final responsibility for the decision to submit for publication.

## Results

In scenario A, we calibrated the model to reflect our historical understanding of the outbreak, with preventive measures implemented. The median number of infections on May 31, 2020, was 1888 (95% prediction interval 1709–2094), representing 4·1% of 46 565 individuals. The results suggest that 24 deaths (16–34), 106 hospital admissions (88–130), and 31 ICU admissions (22–45) occurred up to this date (table 3). By comparing this scenario with scenario B, the counterfactual scenario with no measures targeted to the homeless population, we estimated avoidance of 21 092 infections (19 777–22 147), 266 deaths (226–301), 1164 hospital admissions (1079–1254), and 338 ICU admissions (305–374; table 3).

**Table 3:** Numbers of infections, deaths, hospital admissions, and ICU admissions, in 46 565 people experiencing homelessness under different scenarios

	**R_0_ in community homeless settings**	**Mixing parameter *m***	**COVID-CARE and COVID-PROTECT**	**Second wave**	**SARS-CoV-2 infections**	Cumulative incidence*	**Deaths**	**Hospital admissions**	**ICU admissions (subset of hospital admissions)**
**First wave scenarios: Feb 1–May 31, 2020**									
Scenario A: first wave; base scenario (preventive measures in place)	Low	Low	Yes	*..*	1888 (1709–2094)	4·1% (3·7–4·5)	24 (16–34)	106 (88–130)	31 (22–45)
Scenario B: first wave; do nothing (counterfactual)	High	High	No	*..*	22 933 (21 747–24 053)	49·3% (46·7–51·7)	289 (251–332)	1272 (1180–1369)	372 (337–407)
Difference between scenarios A and B	*..*	*..*	*..*	*..*	21 092 (19 777–22 147)	45·3% (42·5–47·6)	266 (226–301)	1164 (1079–1254)	338 (305–374)
**No second wave scenarios: June 1, 2020–Jan 31, 2021**									
Scenario C: no second wave; retain measures	Low	Low	Yes	No	1025 (856–1201)	6·3% (5·7–6·9)	20 (12–29)	79 (61–98)	23 (14–32)
Scenario D: no second wave; lift measures	High	High	No	No	12 151 (10 718–13 349)	30·1% (27·2–32·8)	184 (151–217)	733 (635–822)	213 (178–251)
Difference between scenarios C and D	*..*	*..*	*..*	*..*	11 168 (9591–12 289)	24·0% (20·6–26·4)	164 (126–197)	653 (554–739)	189 (153–233)
Scenario E: no second wave; lift measures except for COVID-CARE and COVID-PROTECT	High	High	Yes	No	8497 (7202–9515)	22·4% (19·3–24·5)	130 (98–157)	517 (425–612)	152 (111–185)
**Second wave scenarios: June 1, 2020–Jan 31, 2021**									
Scenario F: sharp second wave; retain measures	Low	Low	Yes	Yes	1754 (1543–1960)	7·8% (7·3–8·5)	31 (21–45)	122 (100–148)	35 (23–47)
Scenario G: sharp second wave; lift measures, reduced mixing with general population	High	Low	No	Yes	13 320 (11 861–14 656)	32·7% (29·7–35·3)	209 (168–245)	814 (717–913)	239 (189–276)
Scenario H: flatter second wave; lift measures, reduced mixing with general population	High	Low	No	Yes (flat)	9946 (8682–11 266)	25·4% (22·8–28·3)	149 (119–178)	603 (516–700)	174 (143–211)

Data are median values from 200 runs with 95% prediction intervals. Expanded descriptions of the scenarios are shown in table 2. In scenarios D, G, and H, COVID-CARE and COVID-PROTECT close on Aug 1, 2020. Scenario comparisons show the median and 95% prediction interval of the difference between individual model runs, and therefore values do not sum exactly. SARS-CoV-2=severe acute respiratory syndrome coronavirus 2. ICU=intensive care unit.

* For all scenarios from June 1, 2020, the cumulative incidence includes the incidence in scenario A (ie, the first wave).

**Figure 2:**
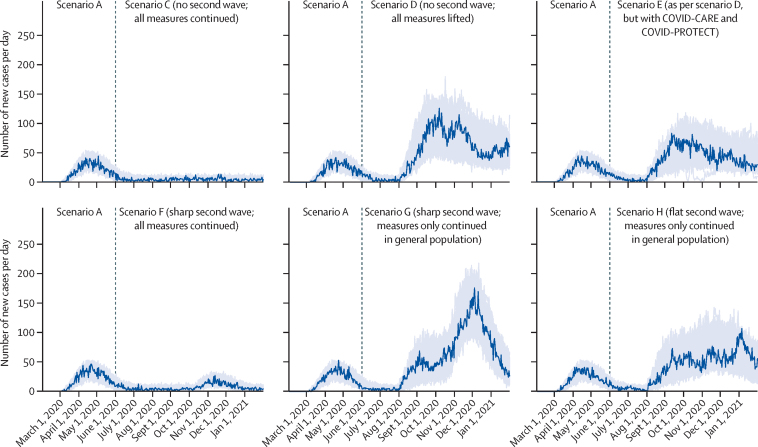
New infections of SARS-CoV-2 among people experiencing homelessness in England, scenarios C, D, E, F, G, and H Scenario A is an estimate of the historical impact of COVID-19 on people experiencing homelessness, and it leads into the future scenarios C–H (table 2). Scenario B is a counterfactual historical scenario that does not lead into the future scenarios, and is therefore not included in the figure. Results from 200 model runs are presented. The dark blue line shows the model run producing the median number of cumulative new cases. SARS-CoV-2=severe acute respiratory syndrome coronavirus 2.

In scenarios C and F, in which preventive measures are continued beyond Aug 1, 2020, there are small numbers of additional infections (ie, similar to those modelled during the first wave) among people experiencing homelessness up to Jan 31, 2021, whether or not there is a second wave in the general population (table 3). By contrast, there are high numbers of infections and deaths in scenarios D, G, and H, in which preventive measures are lifted in August (table 3). In the case of a sharp second wave in the general population (scenario G), the model suggests a sharp peak in infections among people experiencing homelessness coinciding with this second wave, with 13 320 additional infections (95% prediction interval 11 861–14 656) occurring by January, 2021, reaching a cumulative incidence of 32·7% (95% prediction interval 29·7–35·3) since February, 2020 (figure 2). If there is no second wave (scenario D) and mixing with the general population resumes, the profile of infections is flatter but has a similar total by the end of the second wave, with 12 151 additional infections (10 718–13 349), reaching a cumulative incidence of 30·1% (27·2–32·8). This suggests that outbreaks in homeless settings could lead to a high overall attack even if incidence in the general population remains low. If there is no second wave, and COVID-PROTECT and COVID-CARE continue throughout the year, cumulative incidence could be reduced to 22·4% (19·3–24·5), representing 8497 additional infections (7202–9515) from June, 2020 (scenario E).

**Figure 3:**
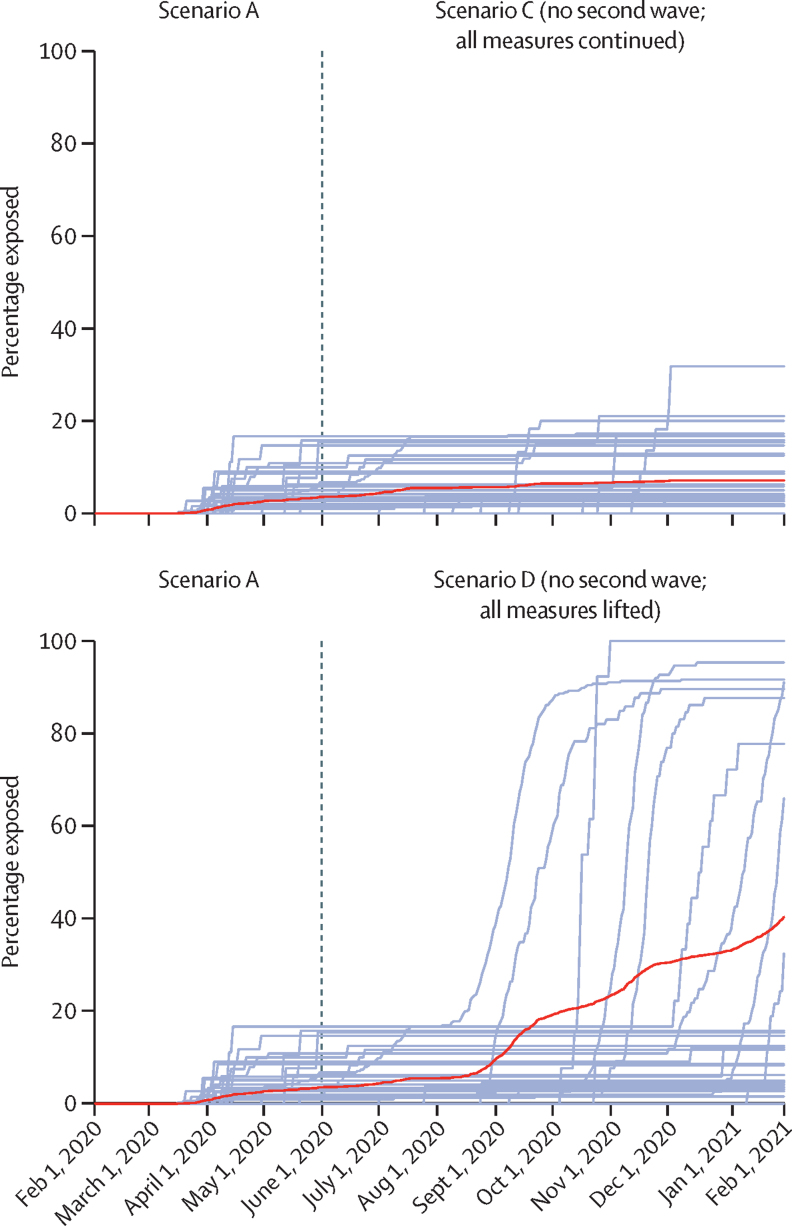
Illustrative timeline of outbreaks in hostels 50 hostels were selected at random for this figure, from a single model run. Each line represents a single hostel, with the total of all 50 hostels shown in red.

In all scenarios, SARS-CoV-2 is modelled as a series of outbreaks within hostels and other homeless settings, with low overall incidence if outbreaks are avoided when cases occur (ie, with prevention measures) and high overall incidence where transmission is not prevented. In figure 3, we illustrate how overall population exposure increases through a series of outbreaks in hostels lasting 1–3 weeks. In scenario A, incidence in the general population is high and hostels are often seeded with cases, but infection control measures mean that few outbreaks occur. In scenario C, where there is no second wave and preventive measures are retained, this situation continues, with lower incidence in the general population. Due to variation in infectiousness of individuals, occasional outbreaks still occur. In scenario D, where there is no second wave and preventive measures are lifted, incidence also remains low in the general population, but infection control measures in hostels are not maintained and outbreaks are more likely to occur when hostels are seeded. These outbreaks sometimes lead to high attack rates within hostels. When comparing number of deaths in scenarios C and D, we find that the combined preventive measures of scenario C could avoid 164 deaths (95% prediction interval 126–197), 89% of those predicted if preventive measures are lifted, between June, 2020, and January, 2021 (table 3). When considering demand for the COVID-PROTECT and COVID-CARE measures, scenario A saw demand for COVID-PROTECT peak at 8609 beds (95% prediction interval 8535–8675) required on a single day, while demand for COVID-CARE peaked at 153 beds (129–178) on a single day. As scenarios with higher incidence of COVID-19 (scenarios D, G, and H) involved closure of these sites, peak demand did not vary substantially between the remaining scenarios.

The univariable sensitivity analyses showed that the number of infections is most sensitive to *R*_0_ in community settings and the dispersion parameter *k* (appendix pp 10–11). The number of deaths is additionally sensitive to the IFR (appendix pp 10–11). The number of cases and deaths is not sensitive to the value of *R*_0_ in COVID-PROTECT hotels, because few infectious individuals are present in these hotels (as transmission depends on pre-symptomatic or asymptomatic individuals being admitted from the community). Other variables had small effects on the number of cases and deaths (appendix pp 10–11). When varying *R*_0_, *k*, the IFR, and the mixing parameter *m* in our multivariable probabilistic sensitivity analysis, we found that maintaining preventive measures in the absence of a second wave (ie, scenario C compared with scenario D) would avoid 10 956 future infections (6376–14 928) and 162 deaths (74–264). Detailed results of sensitivity analyses are provided in the appendix (pp 10–11).

## Discussion

During the first wave of COVID-19 in England, our modelling suggests that people experiencing homelessness were protected by interventions in the general population, infection control in hostels, and closing of dormitory-style accommodation. Our results suggest that 266 deaths were avoided in the first wave, and a further 164 deaths could be avoided if these measures are continued until January, 2021, and potentially more if there is a second wave of COVID-19 in the general population. Even if incidence of COVID-19 remains low in the general population, relaxing measures in hostels and night shelters could lead to outbreaks and a high overall attack rate amongst homeless people.

Outbreaks of COVID-19 in homeless settings have been reported in the USA. In a homeless shelter in Boston, all residents were offered viral testing following identification of symptomatic cases, and 147 (36%) of 408 were positive.^2^ Similar mass screening was done in shelters in Seattle, San Francisco, and Atlanta,^1^, ^31^ with high prevalence in shelters that reported cases before screening. Many cases were asymptomatic, leading to recommendations for universal testing in homeless settings to inform outbreak control.^32^ A shelter in Hamilton, Canada, has increased bed spacing, provided rapid testing for symptomatic residents, and isolated residents with positive or pending results.^33^ This appears to have been successful, with eight confirmed cases of COVID-19 among residents and staff but only one documented secondary case. These experiences suggest that dormitory-style accommodation is susceptible to outbreaks, and preventing outbreaks requires intensive infection control and restructured sleeping arrangements.

In England, homeless people living in hostels or in COVID-PROTECT have been offered testing when symptomatic, and occasionally mass screening exercises have been undertaken. We are not aware of any outbreaks in these settings to date. The results of our model suggest that closing of dormitory-style accommodation and increased infection control in single-room accommodation might have contributed to the absence of outbreaks.

To our knowledge, this is the first study that models SARS-CoV-2 transmission in a homeless population. We modelled outbreaks within real hostels and night shelters by using data collected in censuses of providers. We included learning about the impact of COVID-19 on this population, in particular the probable low number of cases during the first wave, and real-world complexities such as self-discharge from specialist accommodation, even when infectious.

There are important limitations to the knowledge and data that informed our model. First, there is uncertainty about the impact of COVID-19 on people experiencing homelessness to date. We used the number of deaths reported in a surveillance exercise in London to estimate the overall attack rate. As the incidence of COVID-19 in the general population of England is thought to be highest in London,^12^, ^34^ incidence among homeless people might also be highest in London. An estimate of cumulative incidence based on surveillance in London could therefore be an overestimate. Outbreak detection and surveillance of homeless settings could be improved by more systematic testing—eg, through antibody studies or regular viral testing of sentinel sites across the country. Second, there is uncertainty about the severity of COVID-19, both in the general population and for people experiencing homelessness. We used a simple assumption that the increased vulnerability of homeless people to COVID-19 would reflect the previously observed increased risk from deaths due to medical causes (ie, excluding drug overdoses, suicides, and homicides). The risk of infection and mortality due to COVID-19 among homeless people could be studied in more detail using linkage to existing datasets of people experiencing homelessness, such as CHAIN in London.^25^ Third, there is uncertainty about longer-term immunity after infection. Our model does not allow for waning immunity, which appears reasonable over several months,^35^, ^36^ but immunity over longer periods is unknown. A modelling study showed that short-term models of SARS-CoV-2, such as those projecting for 12 months, are unlikely to be sensitive to different assumptions about reinfection.^37^ Fourth, there is uncertainty in modelling parameters—in particular, *k* (the dispersion in the number of expected secondary cases in a susceptible population) and *R*_0_ in community homeless settings. The multivariable probabilistic sensitivity analysis suggested that preventive measures could still avoid 74 deaths and more than 6000 infections, even if the transmission of SARS-CoV-2 and the IFR are lower than expected. Finally, there is uncertainty about the size and structure of the homeless population. We used the best available estimates of the size of the homeless population, but there is still large uncertainty. In England, as in other countries, there are many hidden homeless people who are sofa-surfing or living in squats or in other insecure settings,^38^ and these groups are difficult to count. COVID-19 might have increased the risk of more severe forms of homelessness among these groups. From the population of people sleeping outside or in night shelters, we estimated a peak demand for COVID-PROTECT of 8609 beds. However, the government reports that 14 610 individuals have been accommodated.^18^ This suggests that either we underestimated the size of the population, or there has been an increase since the start of the pandemic in the number of people sleeping rough (or at risk of sleeping rough).

There are also limitations to our modelling methodology. First, we assumed no mixing between subgroups. Outside of the context of the pandemic, there is churn between hostels, night shelters, and street sleeping. In the context of the pandemic, the modelling of homeless settings as closed groups might be reasonable as many of these settings are not accepting new entrants. Second, we did not vary the degree of infectiousness or the duration of disease states by the severity of disease, nor incorporate changing infectiousness over time. Third, we treated the homeless population as static, when it is likely that people continually enter and exit, and the pandemic's wider impacts might have increased the number newly experiencing homelessness. Finally, we modelled step changes in mixing with the general population and the transmissibility of SARS-CoV-2 in homeless settings, when these factors are likely to have changed more gradually, with restrictions sequentially imposed and lifted.

The two main implications for practice are that night shelters should not be re-opened while there is sustained transmission of SARS-CoV-2 in the community, and that heightened infection control measures in hostels should be continued even when incidence of COVID-19 is low in the general population. People who were previously sleeping in night shelters in England are mostly accommodated in commercial hotels. The ongoing accommodation and support of this population will present challenges, but our modelling suggests that outbreaks of COVID-19 could otherwise occur, leading to deaths in this population and potential transmission to other populations. COVID-19 provides an imperative to provide housing for a population that has been underserved for many years. More sustainable housing is also likely to provide health and social benefits beyond a reduction in COVID-19 risk.^39^

There is wide uncertainty in the impact of COVID-19 on people experiencing homelessness. However, there is potential for outbreaks in hostels and night shelters without preventive interventions, and a large number of deaths can be avoided by maintaining the additional support that has so far been provided in England.

## Data sharing

All analysis code and data are publicly available online. This study is a simulation and there are no individual participant data.
