# Supplementary material for: COVID-19 among people experiencing homelessness in England: a modelling study
Source: Lancet Respir Med. 2020 Dec;8(12):1181–91. doi: 10.1016/S2213-2600(20)30396-9 (PMC7511167; doi:10.1016/S2213-2600(20)30396-9)
Supplement: Supplementary appendix [file mmc1.pdf]

THE LANCET
Respiratory Medicine

Supplementary appendix

This appendix formed part of the original submission and has been peer reviewed. We post it as supplied by the authors.

Supplement to: Lewer D, Braithwaite I, Bullock M, et al. COVID-19 among people experiencing homelessness in England: a modelling study. *Lancet Respir Med* 2020; published online Sept 23. http://dx.doi.org/10.1016/S2213-2600(20)30396-9.

Supplementary Information

1. Size and structure of homeless population 2
2. Impact of COVID-19 on the homeless population 5
3. Disease severity and infection fatality rates 7
4. Incidence of SARS-CoV-2 in the general population 9
5. Results of sensitivity analyses 10
6. Detailed flow-chart and state transition equations 13
7. Table of key assumptions 16
8. Studies identified in the literature search (reported in 'research in context' panel) 18
9. Calibration of general population mixing parameter 19
10. References for supplementary information 20

1. Size and structure of homeless population

Our target population was all individuals who were living in homeless hostel accommodation, sleeping rough (sleeping outside), or sleeping in night-shelters in England in March 2020.

i. **People living in homeless hostel accommodation**. We used a census of hostels for single homeless people conducted by Homeless Link,[1] which showed that in early 2020 there were 35,817 bed across 1,065 accommodation projects in 2019 (mean size 33.6 beds, median 21). Occupancy of these hostels is high, with hostels reporting only 784 empty beds on one night in 2018, so for simplicity we used the number of beds as an estimate of the number of residents.

ii. **People sleeping rough**. We estimated the number of people sleeping rough at one point in time using official counts conducted by the UK government on one night in Autumn 2019 (4,266 individuals identified),[2] plus an estimate of case ascertainment (the proportion of the true rough sleeping population identified by this exercise). Case ascertainment was estimated for London based on the CHAIN database,[3] which records rough sleepers identified by street outreach teams over the course of a year. In 2018/19, 2,080 'long-term' rough sleepers were identified, plus 6,775 other individuals who had not been seen in the previous year ('short-term'). We assumed that the mean duration of rough sleeping for the short-term population was 6 weeks, suggesting there would be 782 (6775 x 6/52) short-term rough sleepers at one point in time. This gave an estimate of 2,862 rough sleepers in London, suggesting that the case ascertainment of the official count in London was 40%. We applied this ratio to other regions in England, giving a total population size of 10,748.

iii. **People sleeping in night-shelters.** We assumed that people sleeping in night-shelters are a subset of people sleeping rough. We used data on night-shelters in London collected by the Greater London Authority, which included 44 night-shelters in London, with a total of 963 beds (median 15, IQR 14-20.5). It is likely that this data excludes some night-shelters, as provision is diverse and some night-shelters are operated by small independent organisations. Based on estimates above, this suggests that 963/2,862 = 33% of people sleeping rough in London are sleeping in night-shelters. We applied this proportion to other regions. For the sizes of night-shelters for the whole of England, we sampled the night-shelter sizes in London with replacement to give the target total population size. The Greater London Authority data also show that hostels and 'other supported accommodation' projects are mostly single room, while night-shelters are mostly dormitory-style accommodation (figure S1).

A summary of our population estimates is shown in table S1.

Figure S1: Homeless accommodation projects in London

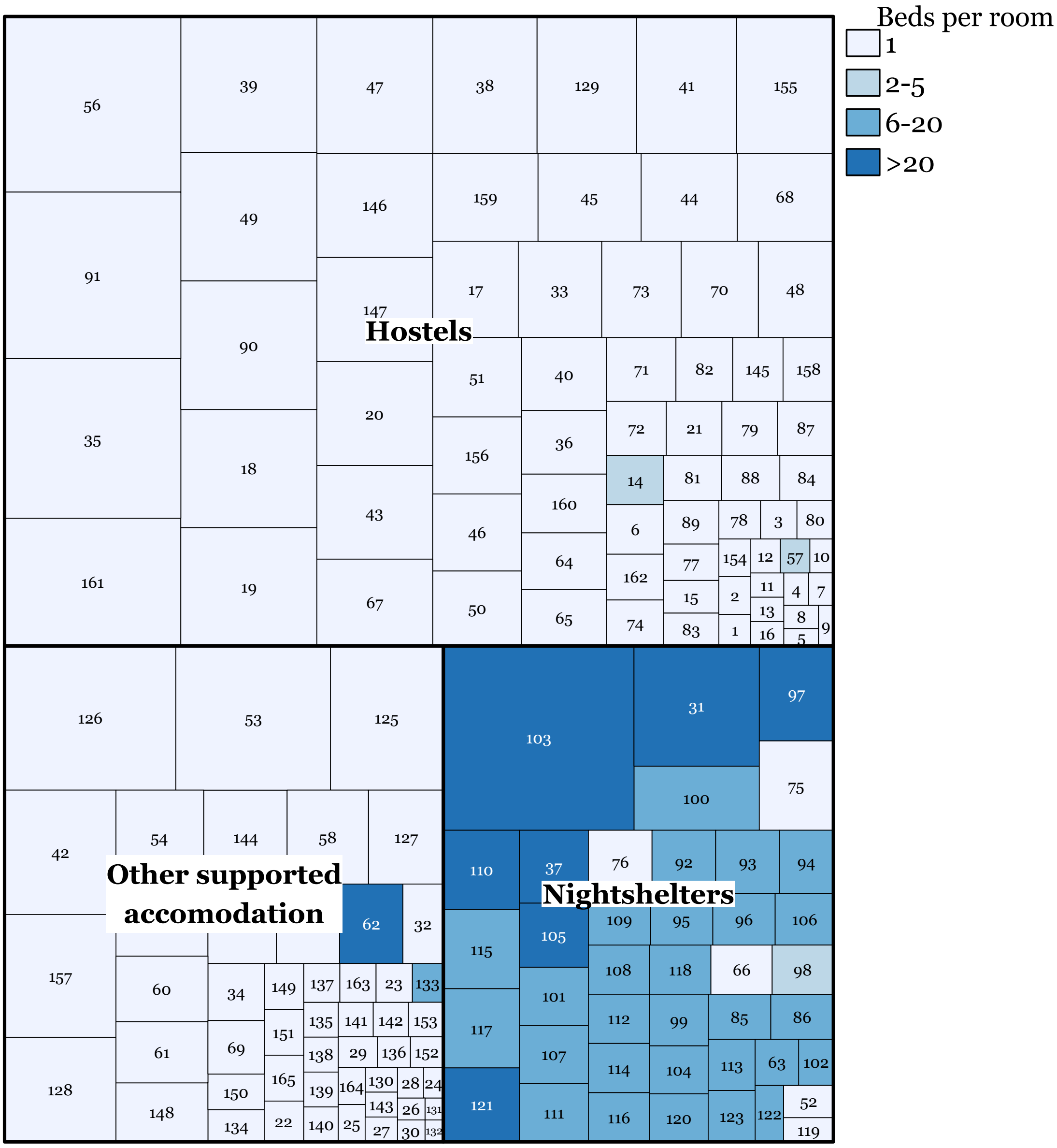

Figure caption: Data is recorded by the Greater London Authority, 1 April 2020. Each square represents one project, with the size of the square representing the total number of beds. The numbers in each square represent the total number of beds including all rooms. The colour of the square represents the number of beds per room (i.e. darker blue projects are dormitory-style accommodation). The total number of beds represented in this figure is 4,624.

Table S1: Estimate of the number of homeless people in England, March 2020, by region

Region	Number of hostel beds[1]	Official count of rough sleepers, 2019[2]	Estimate of rough sleepers	Sleeping in night-shelters	Total population estimate
East	4,009	458	1,154	388	5,163
East Midlands	2,626	305	768	258	3,394
London	9,671	1,136	2,862	963	12,533
North East	1,400	67	169	57	1,569
North West	3,342	349	879	296	4,221
Other	184	0	0	0	0
South East	4,124	900	2,267	763	6,391
South West	4,177	490	1,234	415	5,411
West Midlands	3,623	319	804	271	4,427
Yorkshire	2,661	242	610	205	3,271
**England**	**35,817**	**4,266**	**10,748**	**3,616**	**46,565**

2. Impact of COVID-19 on the homeless population

We identified four sources:

- A surveillance exercise led by the UCL Collaborative Centre for Inclusion Health, in which homeless accommodation projects (including existing hostels and COVID-PROTECT hotel sites) provided weekly reports including numbers of symptomatic residents and deaths due to COVID-19. These projects had a total of 6,075 residents. Between 1 March and 16 June 2020, there were four deaths among these residents (unpublished data collected by authors MB and ACH). Assuming an infection fatality ratio (IFR) of 1.62% (see below), this suggests 247 cases, or a cumulative incidence of 4.1%. Confidence intervals can be estimated using a Poisson method. The 95% confidence limits of a count of 4 deaths are 1.1 and 10.2 deaths, which translates to a confidence interval in the cumulative incidence of 1.1%-10.4%. Note that this does not account for uncertainty in the IFR.

- PCR testing conducted in homeless settings in London, with results collated by UCLH. Between 30 March and 25 May 2020, 842 tests were recorded, of which 28 (3.3%) were positive (unpublished data collected by author AS). We were not able to estimate prevalence or cumulative incidence of infection based on this data because some tests were done following active symptom reporting. The data did suggest that infections in the homeless population followed the same trend as infections in the general population, peaking in early April (see figure below). In addition, no major outbreaks were identified in homeless settings.

- Data published by the Office for National Statistics that shows the number of deaths involving COVID-19 where death registration data indicated that the person was homeless at the time of death.[4] Homelessness was identified if the place of residence or place of death was ‘no fixed abode’ or a known homeless shelter (including COVID-PROTECT and COVID-CARE sites) and the decedent was aged under 75. This data reports 16 deaths in England up to 26 June 2020. This data does not have a denominator and therefore cannot be used to estimate infection rates.

- Analysis by Public Health England found that of all confirmed cases reported by 13 May 2020, 67 ‘no fixed abode’.[5] This value is difficult to interpret because the denominator is unclear and the ‘no fixed abode’ code may be applied in different circumstances.

Figure S2: SARS-CoV-2 infections identified among people experiencing homelessness in London, based on PCR testing done by the UCLH 'Find and Treat' team. In the second plot, tests done in the same settings are surrounded by a solid rectangle. Larger numbers of tests done in the same setting represent mass screening. In the first chart, positive PCR tests for the whole of London are reported by Public Health England.[6] Dates are in 2020.

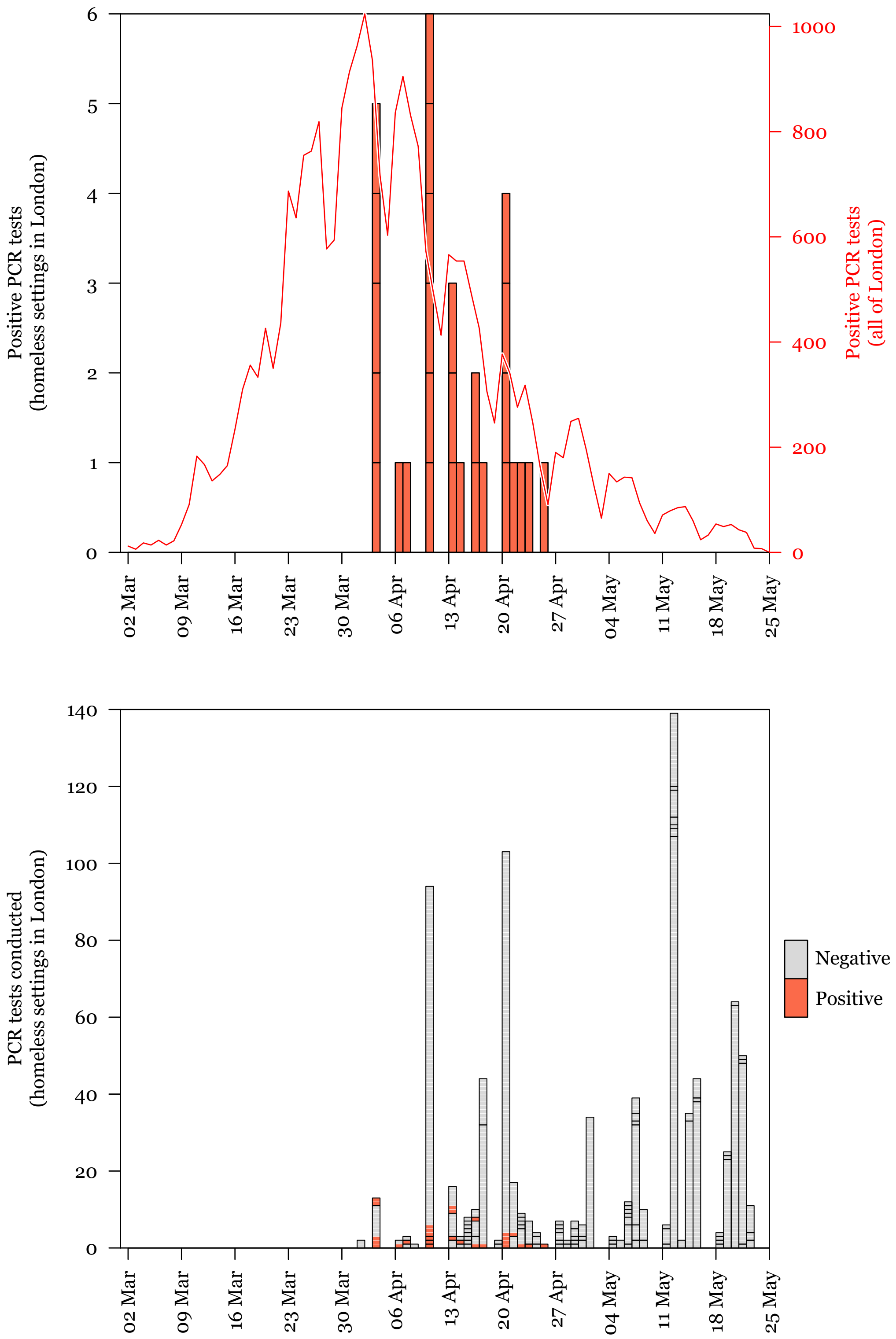

3. Disease severity and infection fatality rates

We estimated the infection fatality rate (IFR) of COVID-19 for homeless people using information about the demographics of the homeless population, infection fatality rates in the general population, and assumptions about the vulnerability of homeless people to more severe disease. We used the following method:

i. **Estimating an age- and sex-weighted IFR for the homeless population, before accounting for health-related vulnerability**. We used age-stratified IFRs estimated in an existing study[7] and calculated age- and sex-stratified IFRs based on male patients having double the risk of death compared to female patients.[8] We applied these IFRs to the age and sex structure of a homeless population to calculate an expected IFR for homeless people of 0.54%, assuming equal chance of infection across age and sex groups. This is a weighted average of the age- and sex-specific IFRs, where the weights are the homeless population. The age and sex profile of the homeless population was provided by the homeless charity St. Mungo's (table S2).

Table S2: age and sex profile of a homeless population (data provided by St. Mungo's), and COVID-19 IFRs and hospitalisation rates in the general population based on an existing modelling study.

	Example homeless population		COVID-19 infection fatality rate			Proportion of COVID-19 cases hospitalised		
Age group	Male	Female	Both sexes[7]	Male	Female	Both sexes[7]	Male	Female
18-29	266	88	0.03%	0.04%	0.02%	1.04%	1.39%	0.69%
30-39	432	142	0.08%	0.11%	0.06%	3.43%	4.60%	2.30%
40-49	422	132	0.16%	0.21%	0.11%	4.25%	5.60%	2.80%
50-59	343	85	0.60%	0.79%	0.40%	8.16%	10.80%	5.40%
60-69	141	20	1.93%	2.57%	1.29%	11.80%	15.80%	7.90%
70-79	46	6	4.28%	5.71%	2.85%	16.60%	22.20%	11.10%
**TOTAL**	**1,650**	**473**						

ii. **Accounting for health-related vulnerability.** Following the observation that risk of death due to COVID-19 is proportional to the risk of death from all-causes[9] (and hence is higher for people who are older or have comorbidities), we used results from cohort studies of mortality rates among homeless people to inform our assumptions about mortality risk from COVID-19. We focused on 'medical' or 'natural' causes of death (such as respiratory diseases, cardiovascular diseases, and cancers), as they are more likely to represent vulnerability to COVID-19 than deaths due to drugs, alcohol, suicide, or homicide. We found two studies reporting mortality due to 'medical' or 'natural' causes, which reported standardised mortality ratios of 3.6[10] and 2.6.[11] We therefore assumed that the COVID-19 IFR for people experiencing homelessness would be three times that of people of the same age and sex in the general population, i.e. 0.54 * 3 = 1.62%.

iii. **Estimating the proportion of cases by severity**. We needed to classify patients by severity to understand how many are symptomatic (and hence would be identified for transfer to COVID-CARE) and how many may require admission to hospital or ICU. We used four disease severity categories: asymptomatic, mild (offered COVID-CARE), moderate (requiring hospital admission) and severe (requiring ICU admission). We used the following process to set the IFRs for each category and the proportion of cases in each category:

a. We made the assumption that 50% of deaths would occur among people who were admitted to ICU. This follows the observation that approximately 30% of deaths among people with confirmed COVID-19 in England were among people admitted to ICU (with 42,461 deaths among people with confirmed COVID-19,[6] and 12,573 ICU admissions, as of 19 June 2020[12]), and we assumed this proportion would be higher among a relatively young homeless population.

b. We assumed an IFR of 45% for patients admitted to ICU.[12,13] We combined this assumption with an overall IFR of 1.62% and the assumption that 50% of deaths occur among patients admitted to ICU to estimate that 1.62% * 50% / 45% = 1.8% of all cases are admitted to ICU.

c. We used an existing estimate of the age- and sex-stratified risk of hospitalisation[7] to estimate that 6.2% of cases among homeless people would require hospitalisation (see table S2). This is a weighted average of age- and sex-specific risks of hospitalisation, using the homeless population as weights.

d. As 1.8% of cases are admitted to ITU, 6.2% - 1.8% = 4.4% are hospitalised without ICU admission.

e. We assumed an IFR of 15% for patients who are hospitalised without ICU, based on published case series.[14,15]

f. We assumed that 40% of cases are asymptomatic, based on studies of intensive testing in confined settings.[16,17]

g. This left 53.8% of cases classified as mild. We deduced an IFR of 0.28% for these cases, to achieve the overall IFR of 1.62%.

These values are summarised in table 1 in the main article.

4. Incidence of SARS-CoV-2 in the general population

Our model uses incidence of SARS-CoV-2 in the general population as a 'background risk' for the homeless population, in combination with a parameter *m* indicating the degree of mixing between the homeless population and the general population. Cases arising from this 'background risk' act as seeds for transmission within homeless settings such as hostels and night-shelters, which are modelled as closed groups.

To estimate incidence of SARS-CoV-2, we used a seroprevalence survey of people living in private households conducted between 26 April and 13 June 2020 that indicated 5.4% had developed antibodies,[18] equating to approximately 3 million cases in England. We drew a simple curve of the daily number of new cases that achieves this number of infections, following the start date, peak, and end dates of the first 'wave', based on COVID-19 PCR testing data.[6] For the purposes of sensitivity analysis, we also created first waves with a 'high profile' and a 'low profile'. For the 'high profile', we used a modelled estimate that 8% of the general population of England has been infected.[19] For the 'low profile', we used a cumulative incidence of 2.8% (5.4% - 8% +5.4%).

For future scenarios, we assumed a 'baseline' of 5,000 infections per day in the absence of large-scale transmission, based on an estimate that there were 4,200 new infections per day between 20 July and 26 July 2020.[20] We created two scenarios of a 'second wave', each with half the total number of infections of the first wave. The first had a 'sharp profile, with the same duration as the first wave and a maximum incidence on 1 November 2020. The second had a 'flatter profile', with three times the duration of the first wave and a maximum incidence also on 1 November 2020.

The code used to generate these scenarios is available at https://github.com/maxeyre/Homeless-COVID-19/blob/master/model/SUPPORTING_general_population_incidence.R (note this code does not need to be run prior to running the model, as the results are already saved in the online repository, and read directly by the model code).

Figure S3: modelled number of daily new SARS-CoV-2 infections in the general population of England

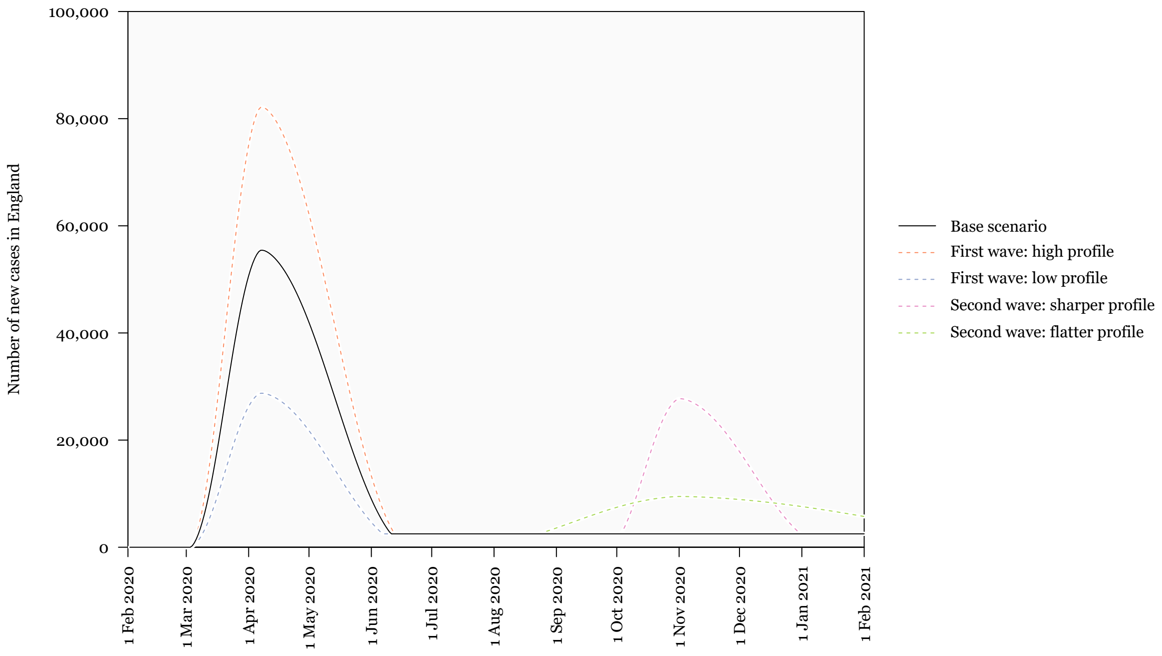

5. Results of sensitivity analyses

Univariable analysis

Table S3: Results of sensitivity analyses. Values of infections and deaths are median values from 200 runs with 95% prediction intervals.

Parameter	Base scenario	Value	Infections	Deaths
Overall infection mortality rate	D	0.54% (low)	12,071 (11,015-13,315)	61 (48-74)
		1.62% (base)	12,151 (10,718-13,350)	184 (151-217)
		2.70% (high)	12,217 (10,795-13,360)	310 (262-350)
R_0 in community homeless settings (the values shown are for hostels; night-shelters are 1.5x these values, and people sleeping rough have 0.75x these values)	D	1.5 (low)	5,952 (4,971-6,871)	91 (70-114)
		2.5 (base)	12,151 (10,718-13,350)	184 (151-217)
		3.5 (high)	15,758 (14,000-16,952)	239 (208-282)
k (dispersion in infectivity)	D	0.1 (low)	5,454 (4,253-6,557)	83 (59-111)
		1 (base)	12,151 (10,718-13,350)	184 (151-217)
		10 (high)	14,732 (13,385-15,777)	227 (186-264)
		No clustering	16,951 (15,784-17,946)	258 (227-289)
First wave cumulative incidence in general population	D (numbers are given for days 1-365)	2.8% (low)	13,578 (12,348-14,923)	204 (173-240)
		5.4% (base)	13,970 (12,602-15,182)	209 (173-241)
		8.0% (high)	14,184 (12,869-15,529)	215 (183-245)
R_0 in COVID-PROTECT	G	0 (low)	7,781 (6,722-8,630)	128 (98-157)
		0.75 (base)	8,498 (7,202-9,515)	130 (98-157)
		2.5 (high)	8,448 (7,460-9,734)	134 (97-162)
Proportion that accept COVID-PROTECT or COVID-CARE when offered	G	0.5 (low)	9,330 (8,126-10,625)	144 (117-176)
		0.8 (base)	8,498 (7,202-9,515)	130 (99-157)
		1 (high)	7,726 (6,653-8,866)	118 (90-151)
Daily risk of self-discharge	G	0% (low)	7,474 (6,419-8,506)	115 (92-143)
		0.59% (base)	8,498 (7,202-9,515)	130 (99-157)
		1.58% (high)	9,343 (7,995-10,362)	142 (114-170)
Mixing with the general population after the first wave	E	0.1 (low)	389 (294-525)	7 (3-13)
		0.5 (base)	1,754 (1,543-1,960)	31 (21-45)
		1 (high)	3,134 (2,860-3,412)	58 (42-75)

Different base scenarios are used (D, E or G) to provide the most relevant comparison. For example, we used scenario G for R_0 in COVID-PROTECT because interventions are open for the full model duration in this scenario. Except for the scenario comparing different first waves, results are reported for 1 June 2020-31 January 2021, for consistency with the main article.

Figure S4: Tornado plot summarising results of sensitivity analysis

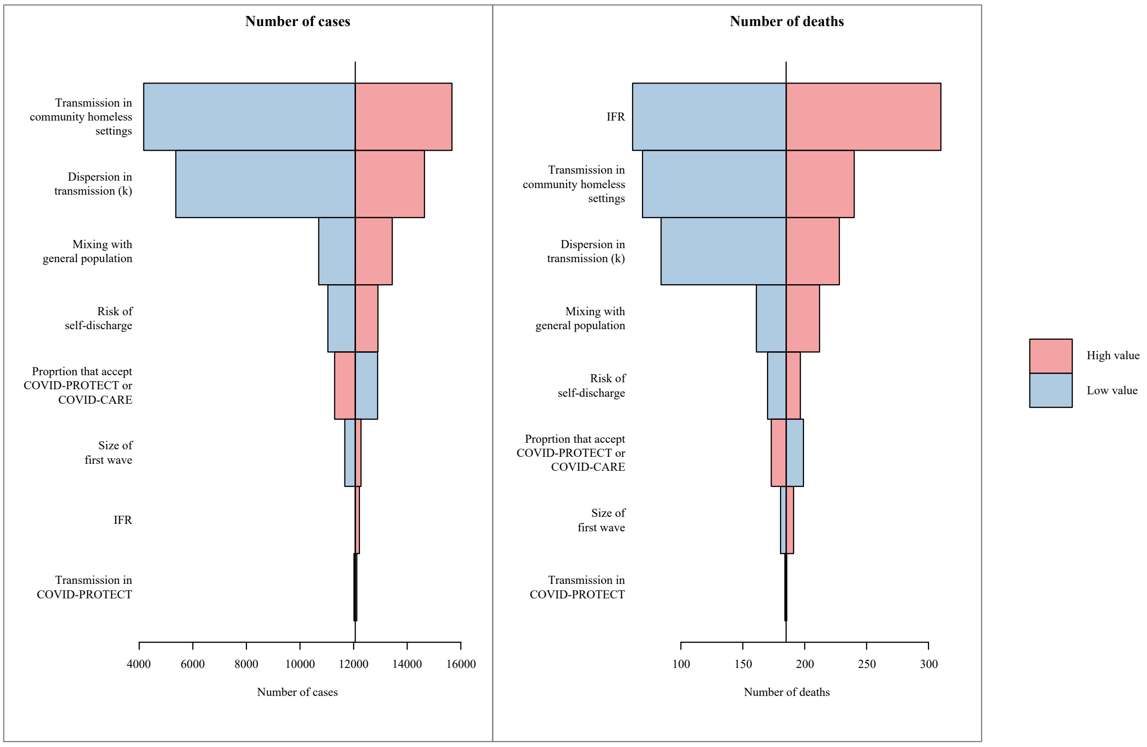

The figure shows the variation from the base scenario with low and high input values. Values on the x-axes refer to sensitivity analyses using scenario D as a baseline (hence the central vertical line corresponds to the median numbers of cases and deaths in scenario D). For other sensitivity analyses using other base scenarios, the chart shows the differences rather than actual values (refer to the table for actual values).

Multivariable analysis

We conducted a Monte-Carlo sensitivity analysis in which key parameters were varied simultaneously. Based on the univariable analysis, we selected three variables that have a large effect on the model results: transmission in community homeless settings, the dispersion parameter *k*, and the IFR. In addition, we included the mixing parameter *m* because it may have important effects in scenarios F and H. We assumed the following distributions for these parameters:

- **Transmission in community homeless settings**: We used a PERT distribution in R_0 for hostels, with minimum = 1.5, mode = 2.5, and maximum = 3.5. People sleeping outside and in night-shelters were assumed to have values of 0.75 and 1.5 times the value for hostels, respectively.

- **Dispersion parameter *k*.** Existing evidence suggests that the credible interval for the median value of *k* in the general population is log-normally distributed (*k* = 0.1, 95% CrI 0.05, 0.2).[21] Our base value of *k* is 1, and we therefore log-transformed these values and shifted them arithmetically by *ln*(0.1) such that the median value is *ln*(k) = 0 (95% CrI -0.69, 0.69). We then sampled values of *ln*(k) from a normal distribution with mean = 0 and standard deviation of 0.69/1.96, then exponentiated.

- **IFR**. We used a PERT distribution in the multiplier described in Supplementary Information 3 with minimum = 1 (i.e. the same mortality risk as people of the same age and sex in the general population), mode = 3, and maximum = 5 (i.e. minimum overall IFR = 0.54%, mode = 1.62%, maximum = 2.70%).

- **Mixing parameter *m*.** Following the calibration detailed in Supplementary Information 9, we assumed a minimum of 0.1 (corresponding to a cumulative incidence of approximately 1% during the first wave), while the 'natural' upper bound for this parameter is 1. We used a PERT distribution with minimum = 0.1, mode = 0.5, and maximum = 1.

We sampled 200 parameter sets from these distributions and ran the scenarios under each parameter set. Results from these analyses are shown in table S4.

Table S4: Results of multivariable sensitivity analysis (median from 200 runs with 95% prediction intervals)

**Scenario**	**SARS-CoV-2 infections**	**Deaths**	**Hospital admissions**	**ITU admissions (subset of hospital admissions)**
First wave scenarios: 1 February 2020 – 31 May 2020				
A: First wave; base scenario (preventative measures in place)	1,890 (768-3,328)	23 (6-52)	107 (43-189)	30 (11-57)
B: First wave; do nothing	22,705 (16,106-27,515)	276 (138-467)	1,250 (844-1,558)	363 (247-457)
Difference between scenarios A and B	20,847 (13,567-26,008)	254 (121-437)	1,153 (726-1,477)	332 (209-434)
No second wave scenarios: 1 June 2020 – 31 January 2021				
C: No second wave; retain measures	1,062 (497-1,573)	19 (7-42)	78 (36-130)	23 (10-42)
D: No second wave; lift measures	11,954 (7,343-15,953)	180 (87-293)	731 (451-958)	210 (128-293)
Difference between scenarios C and D	10,956 (6,376-14,928)	162 (74-264)	650 (371-893)	186 (104-271)
E: No second wave; lift measures except for COVID-CARE and COVID-PROTECT	8,423 (4,756-11,676)	122 (54-240)	511 (295-710)	148 (83-216)
Second wave scenarios: 1 June 2020 – 31 January 2021				
F: Sharp second wave; retain measures	1,750 (755-2,837)	28 (10-65)	123 (53-198)	35 (14-63)
G: Sharp second wave; lift measures, reduced mixing with general population	12,170 (6,979-19,474)	180 (93-387)	741 (419-1,227)	214 (117-348)
H: Flatter second wave; lift measures, reduced mixing with general population	9,104 (5,012-15,480)	137 (59-296)	550 (287-979)	159 (82-282)

Table caption: the shaded rows comparing scenarios show the median and 95% prediction interval of the difference between individual model runs, and therefore values shown in the table do not sum exactly.

6. Detailed flow-chart and state transition equations

Figure S5: Detailed flow-chart of state transitions. COVID-PROTECT is only offered to individuals sleeping in night-shelters or sleeping rough, so where s=3 (hostels), all CPin and CPout flows equal zero.

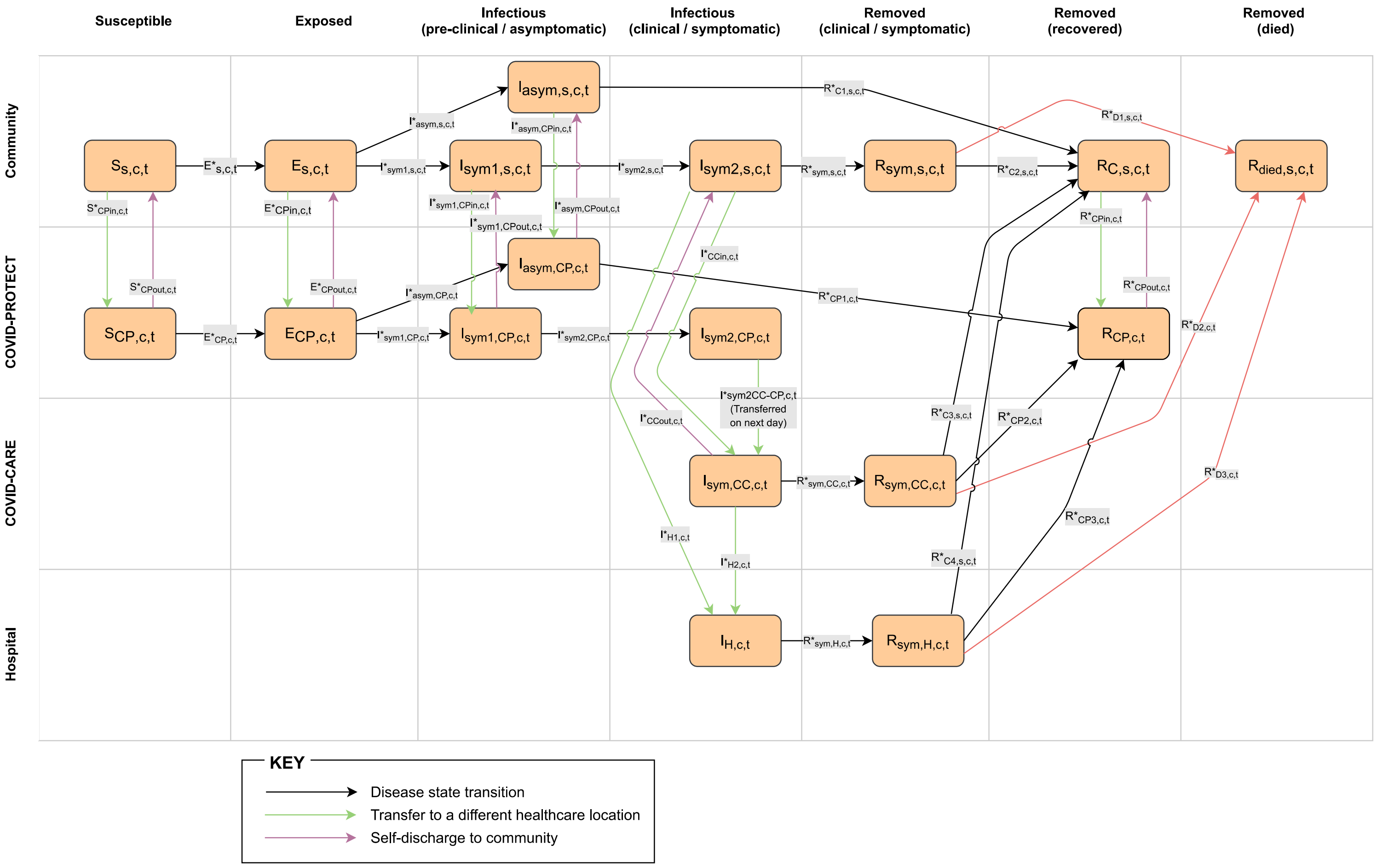

State transition equations

We define the daily flow of individuals between each compartment for every population sub-group, s, representing the three community population groups of rough sleepers (s=1), night-shelters (s=2) and hostels (s=3), and each cluster, c, within each sub-group at time, t. Flows between compartments are denoted with a superscript '*'. The COVID-PROTECT, COVID-CARE and hospital population sub-groups are denoted with the subscripts 'CP', 'CC' and 'H', respectively. Transfers from the community to COVID-PROTECT and COVID-CARE are denoted with 'CPin' and 'CCin', while self-discharges from these healthcare locations to the community are denoted with 'CPout' and 'CCout'. When individuals return from healthcare locations to the community, they return to their original population sub-group and cluster.

For rough sleepers and night-shelters:

$$S_{s,c,t+1}=S_{s,c,t}-E^{*}_{s,c,t}-S^{*}_{CPin,c,t}+S^{*}_{CPout,c,t}$$
$$E_{s,c,t+1}=E_{s,c,t}+E^{*}_{s,c,t}-I^{*}_{sym1,s,c,t}-I^{*}_{asym,s,c,t}-E^{*}_{CPin,c,t}+E^{*}_{CPout,c,t}$$
$$I_{asym,s,c,t+1}=I_{asym,s,c,t}+I^{*}_{asym,s,c,t}-I^{*}_{asym,CPin,c,t}+I^{*}_{asym,CPout,c,t}-R^{*}_{C1,s,c,t}$$
$$I_{sym1,s,c,t+1}=I_{sym1,s,c,t}+I^{*}_{sym1,s,c,t}-I^{*}_{sym1,CPin,c,t}+I^{*}_{sym1,CPout,c,t}-I^{*}_{sym2,s,c,t}$$
$$I_{sym2,s,c,t+1}=I_{sym2,s,c,t}+I^{*}_{sym2,s,c,t}-I^{*}_{CCin,c,t}+I^{*}_{CCout,c,t}-I^{*}_{H1,c,t}-R^{*}_{sym,s,c,t}$$
$$R_{sym,s,c,t+1}=R_{sym,s,c,t}+R^{*}_{sym,s,c,t}-R^{*}_{C2,c,t}-R^{*}_{D1,s,c,t}$$
$$R_{C,s,c,t+1}=R_{C,s,c,t}+R^{*}_{C1,c,t}+R^{*}_{C2,c,t}+R^{*}_{C3,c,t}+R^{*}_{C4,c,t}-R^{*}_{CPin,c,t}+R^{*}_{CPout,c,t}$$
$$R_{died,s,c,t+1}=R_{died,s,c,t}+R^{*}_{D1,s,c,t}+R^{*}_{D2,c,t}+R^{*}_{D3,c,t}$$

For COVID-PROTECT, COVID-CARE and hospital:

$$S_{CP,c,t+1}=S_{CP,c,t}-E^{*}_{CP,c,t}+S^{*}_{CPin,c,t}-S^{*}_{CPout,c,t}$$
$$E_{CP,c,t+1}=E_{CP,c,t}+E^{*}_{CP,c,t}-I^{*}_{sym1,CP,c,t}-I^{*}_{asym,CP,c,t}+E^{*}_{CPin,c,t}-E^{*}_{CPout,c,t}$$
$$I_{asym,CP,c,t+1}=I_{asym,CP,c,t}+I^{*}_{asym,CP,c,t}+I^{*}_{asym,CPin,c,t}-I^{*}_{asym,CPout,c,t}-R^{*}_{CP1,s,c,t}$$
$$I_{sym1,CP,c,t+1}=I_{sym1,CP,c,t}+I^{*}_{sym1,CP,c,t}+I^{*}_{sym1,CPin,c,t}-I^{*}_{sym1,CPout,c,t}-I^{*}_{sym2,CP,c,t}$$
$$I_{sym2,CP,c,t+1}=I_{sym2,CP,c,t}+I^{*}_{sym2,CP,c,t}-I^{*}_{sym2CPCC,c,t}$$
$$I_{sym,CC,c,t+1}=I_{sym,CC,c,t}+I^{*}_{CCin,c,t}-I^{*}_{CCout,c,t}+I^{*}_{sym2CPCC,c,t}-I^{*}_{H2,c,t}-R^{*}_{sym,CC,c,t}$$
$$I_{H,c,t+1}=I_{H,c,t}+I^{*}_{H1,c,t}+I^{*}_{H2,c,t}-R^{*}_{sym,H,c,t}$$
$$R_{sym,CC,c,t+1}=R_{sym,CC,c,t}+R^{*}_{sym,CC,c,t}-R^{*}_{C3,c,t}-R^{*}_{CP2,c,t}-R^{*}_{D2,c,t}$$
$$R_{sym,H,c,t+1}=R_{sym,H,c,t}+R^{*}_{sym,H,c,t}-R^{*}_{C4,c,t}-R^{*}_{CP3,c,t}-R^{*}_{D3,c,t}$$
$$R_{CP,c,t+1}=R_{CP,c,t}+R^{*}_{CP1,c,t}+R^{*}_{CP2,c,t}+R^{*}_{CP3,c,t}+R^{*}_{CPin,c,t}-R^{*}_{CPout,c,t}$$

Where:

$$E^{*}_{s,c,t}\sim\text{Binomial}(S_{s,c,t},\ p_{s,c,t})$$
$$E^{*}_{CP,c,t}\sim\text{Binomial}(S_{CP,c,t},\ p_{CP,c,t})$$

These random variables are binomially distributed with the following probabilities:

$$p_{s,c,t}=1-\left(1-FOI_{s,c,t}\right)(1-m)$$
$$p_{CP,c,t}=1-\left(1-FOI_{CP,c,t}\right)(1-m)$$

All flows from either the rough sleeper (s=1) or night-shelter (s=2) community sub-groups to healthcare locations are binomially distributed with probability p_{accept} and $p_{discharge}$ for transfer to the location and self-

discharge, respectively. However, individuals in the hostel sub-group (s=3) cannot be transferred into COVID-PROTECT and $p_{accept}=0$ for ‘CPin’ flows from hostels. The number of days that each individual spends in each Exposed, Infectious and Removed state is drawn from discrete gamma distributions (these are independent of which community sub-group or healthcare location each individual is in) as described in Table S2. Flows to hospital are binomially distributed with probabilities defined in table 2 of the main article. Flows to Removed (died) are binomially distributed with probabilities also defined in table 2, and all other individuals moving to the Removed (recovered) compartment. The transfer of symptomatic infectious individuals from COVID-PROTECT to COVID-CARE, $I^{*}_{sym2CPCC,c,t}$, is not a random variable and follows a regular timetable with individuals moving from $I_{sym2,CP,c,t}$ to $I_{sym,CC,c,t}$ after one day.

7. Table of key assumptions

Table S5: Key assumption used in the model

Assumption	Value	Source
Cumulative incidence of SARS-CoV-2 in the general population at end of May 2020	5.4%	Coronavirus (COVID-19) Infection Survey pilot: 18 June 2020[18]
Incidence of SARS-CoV-2 in the general population between June 2020 and January 2021	Baseline of 5,000 infections per day in the absence of a 'second wave', or an additional 2.7% of the population is infected if there is a 'second wave'	Based on an estimate that there were 4,200 new infections per day between 20 July and 26 July 2020.[20]
R_0 in community homeless settings and COVID-PROTECT, with containment measures	0.75	Average transmission in the general population when containment measures are in place (with the average number of secondary cases estimated at 0.7-0.9 in June 2020).[22]
R_0 in community homeless settings, in the absence of containment measures	2.5 in hostels<br>1.875 for people sleeping outside<br>3.75 for night-shelters	2.5 is based on estimated values for the general population without strong containment measures[23] and the average number of secondary cases estimated on the Diamond Princess.[24] People sleeping outside are likely to be at lower risk (though still often mix in groups), and are therefore assigned 0.75x this level of transmission, while people sleeping in night-shelters are likely to have higher risk and are therefore assigned 1.5x this level of transmission.
k (dispersion parameter for the expected number of secondary cases per individual, in a susceptible population)	1	The number expected secondary cases is modelled as a negative binomial distribution across individuals, where the mean is R_0 and *k* is the dispersion parameter. Where $k = \infty$; the distribution is equivalent to a Poisson distribution. Evidence suggests a high degree of variation in the number of secondary transmissions of SARS-CoV-2 in the general population, with an estimated median *k* of 0.1.[21] We assumed that the number of secondary cases would be less dispersed among homeless people than in the general population, because homeless people are a specific subgroup with shared characteristics. We therefore set *k* to 1.
Overall infection fatality rate	1.62%	See workings above
Latent period (exposed)<br><br>Pre-clinical (asymptomatic) infectious period<br><br>Clinical (symptomatic) infectious period<br><br>Duration of hospitalisation	gamma(u = 4 days, k = 4)<br><br>gamma(u = 1.5 days, k = 4)<br><br>gamma(u = 3.5 days, k = 4)<br><br>gamma(u = 8 days, k = 8)	Following a model of COVID-19 in the general population of the UK.[23] We used a discrete gamma distribution such that individuals had integer values of these durations, in days. See figure below for an example of disease durations used in the model.
Infectious period d_i	Mean 5 days	Derived; pre-clinical (asymptomatic) infectious period plus clinical (symptomatic) infectious period
Force of infection	$1 - \exp(I_s/N_s * 1/d_i * SC_s)$<br>$I_s$ = number of infectious individuals in the subgroup<br>N_s = number of individuals in the subgroup	Derived from other inputs

Assumption	Value	Source
	SC_s = mean number of expected secondary cases for infectious individuals in the subgroup	
m (mixing with the general population)	Depending on scenario (see table 2 in the main article)	Assumption
Proportion of people sleeping outside who accept COVID-PROTECT	80%	Based on programme experience and observations from local authorities that some people are still sleeping outside, but in much reduced numbers
Proportion of people with confirmed COVID-19 who accept COVID-CARE	80%	Based on programme experience
Daily risk of self-discharge from COVID-PROTECT or COVID-CARE	0.59%	Based on the risk of self-discharge during hospital admissions (which was 8% over 2 weeks in a study of 3,222 homeless inpatients in England[25]). 0.59% = 1 - (1-0.08)^(1/14)
Duration over which people are moved into COVID-PROTECT	28 days	Based on programme experience

Figure S6: Example of disease durations. Durations follow a discrete gamma distribution with means and dispersion values specified in table S4.

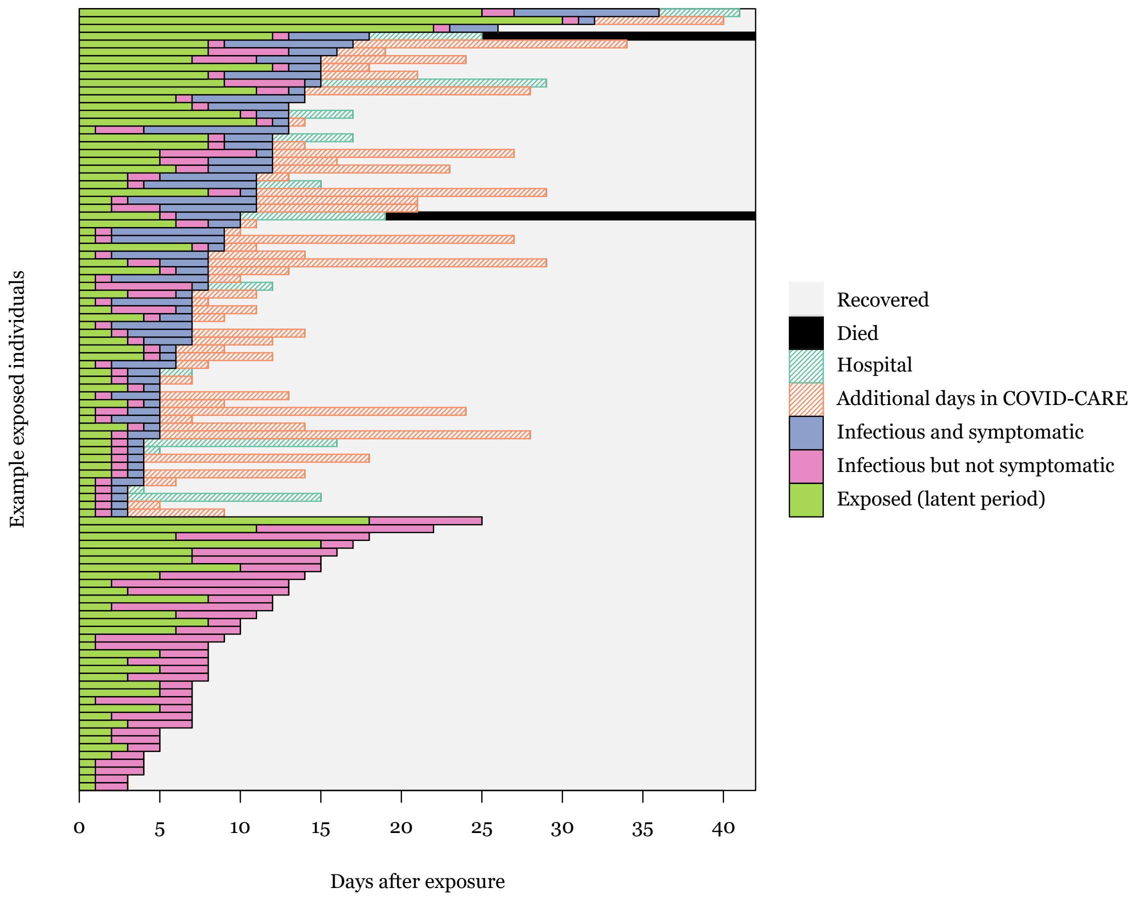

8. Studies identified in the literature search (reported in 'research in context' panel)

The search strategy is described in the 'research in context' panel. We found 14 relevant studies and documents.[26–39]

9. Calibration of general population mixing parameter

We ran Scenario A varying the general population mixing parameter *m* between 0.1 and 1 at increments of 0.05. At each increment, we ran the model 200 times and reported the median cumulative incidence of SARS-CoV-2 infection at 31 May 2020.

Figure S7: Mixing parameter *m* and median cumulative incidence at 31 May 2020

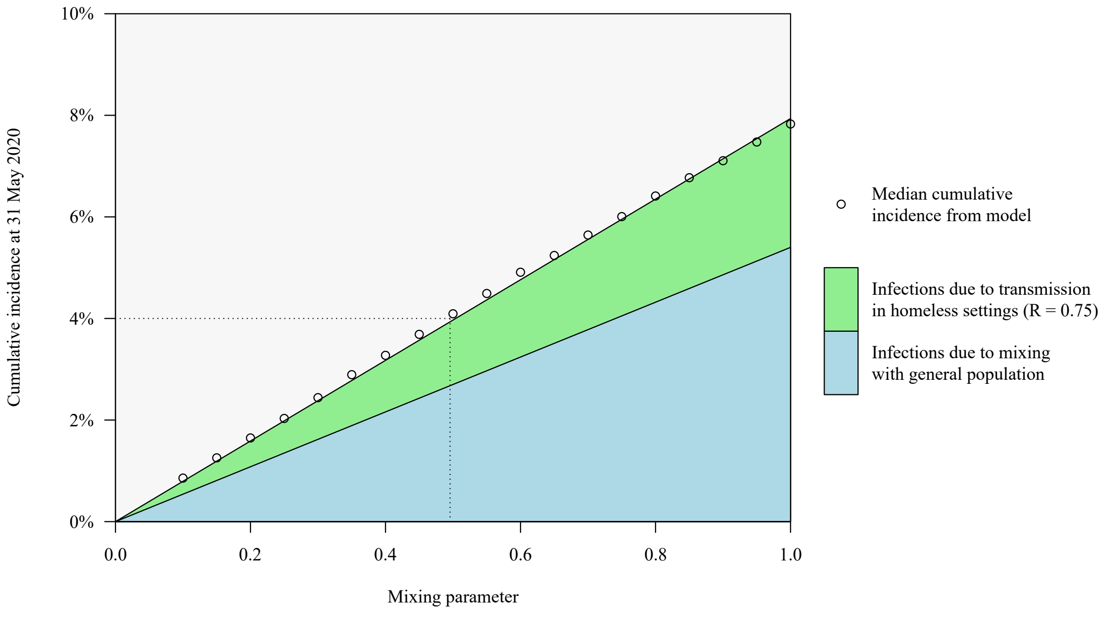

10. References for supplementary information

1 Homeless Link. Support for single homeless people in England, Annual Review 2018. 2019. https://www.homeless.org.uk/sites/default/files/site-attachments/Annual%20Review2018.pdf (accessed April 1, 2020).

2 Ministry of Housing, Communities & Local Government. Rough sleeping snapshot in England: autumn 2019. 2020. https://www.gov.uk/government/statistics/rough-sleeping-snapshot-in-england-autumn-2019 (accessed April 1, 2020).

3 Greater London Authority. Rough sleeping in London (CHAIN reports). 2019. https://data.london.gov.uk/dataset/chain-reports (accessed April 24, 2020).

4 Office for National Statistics. Coronavirus and deaths of homeless people, England and Wales: deaths registered up to 26 June 2020. 2020. https://www.ons.gov.uk/peoplepopulationandcommunity/birthsdeathsandmarriages/deaths/articles/coronavirusanddeathsofhomelesspeopleenglandandwalesdeathsregisteredupto26june2020/2020-07-10 (accessed Aug 2, 2020).

5 Public Health England. Disparities in the risk and outcomes from COVID-19. 2020. https://assets.publishing.service.gov.uk/government/uploads/system/uploads/attachment_data/file/890258/disparities_review.pdf (accessed June 8, 2020).

6 Department for Health and Social Care. Coronavirus (COVID-19) in the UK. 2020. https://coronavirus.data.gov.uk/ (accessed June 12, 2020).

7 Verity R, Okell LC, Dorigatti I, *et al.* Estimates of the severity of coronavirus disease 2019: a model-based analysis. *Lancet Infect Dis* 2020; **20**: 669–77. DOI:10.1016/S1473-3099(20)30243-7.

8 Bacon S, Bates C, Morton CE, *et al.* OpenSAFELY: factors associated with COVID-19-related hospital death in the linked electronic health records of 17 million adult NHS patients. *medRxiv* 2020. DOI:10.1101/2020.05.06.20092999.

9 Spiegelhalter D. How much ‘normal’ risk does COVID-19 represent? https://wintoncentre.maths.cam.ac.uk/news/how-much-normal-risk-does-covid-19-represent/ (accessed April 24, 2020).

10 Stenius-Ayoade A, Haaramo P, Kautiainen H, Gissler M, Wahlbeck K, Eriksson JG. Mortality and causes of death among homeless in Finland: a 10-year follow-up study. *J Epidemiol Community Health* 2017; **71**: 841–8. DOI:10.1136/jech-2017-209166.

11 Nordentoft M. 10 year follow up study of mortality among users of hostels for homeless people in Copenhagen. *BMJ* 2003; **327**: 81–0. DOI:10.1136/bmj.327.7406.81.

12 ICNARC. ICNARC report on COVID-19 in critical care 19 June 2020. 2020. https://www.icnarc.org/Our-Audit/Audits/Cmp/Reports (accessed June 12, 2020).

13 Doherty A, Harrison E, Green C, *et al.* Features of 16,749 hospitalised UK patients with COVID-19 using the ISARIC WHO Clinical Characterisation Protocol. *COVID-19 SARS-CoV-2 Prepr MedRxiv BioRxiv* 2020. DOI:10.1101/2020.04.23.20076042.

14 Richardson S, Hirsch JS, Narasimhan M, *et al.* Presenting Characteristics, Comorbidities, and Outcomes Among 5700 Patients Hospitalized With COVID-19 in the New York City Area. *JAMA* 2020; **323**: 2052. DOI:10.1001/jama.2020.6775.

15 Chen N, Zhou M, Dong X, *et al.* Epidemiological and clinical characteristics of 99 cases of 2019 novel coronavirus pneumonia in Wuhan, China: a descriptive study. *The Lancet* 2020; **395**: 507–13. DOI:10.1016/S0140-6736(20)30211-7.

16 Day M. Covid-19: identifying and isolating asymptomatic people helped eliminate virus in Italian village. *BMJ* 2020; **368**. DOI:10.1136/bmj.m1165.

17 Emery JC, Russell TW, Liu Y, Hellewell J, Pearson CA. The contribution of asymptomatic SARS-CoV-2 infections to transmission - a model-based analysis of the Diamond Princess outbreak. 2020. https://cmmid.github.io/topics/covid19/reports/Emery_Transmission%20from%20asymptomatic%20SARS-CoV-2.pdf (accessed Aug 17, 2020).

18 Office for National Statistics. Coronavirus (COVID-19) Infection Survey pilot: 18 June 2020. 2020. https://www.ons.gov.uk/peoplepopulationandcommunity/healthandsocialcare/conditionsanddiseases/bulletins/coronaviruscovid19infectionsurveypilot/18june2020 (accessed June 19, 2020).

19 Birrell P, Blake J, van Leeuwen E, Joint PHE Modelling Cell, MRC Biostatistics Unit COVID-19 Working Group, De Angelis D. COVID-19: nowcast and forecast. 2020. https://www.mrc-bsu.cam.ac.uk/now-casting/ (accessed June 19, 2020).

20 Office for National Statistics. Coronavirus (COVID-19) Infection Survey pilot: England, 31 July 2020. 2020. https://www.ons.gov.uk/peoplepopulationandcommunity/healthandsocialcare/conditionsanddiseases/bulletins/coronaviruscovid19infectionsurveypilot/31july2020 (accessed Aug 4, 2020).

21 Endo A, Centre for the Mathematical Modelling of Infectious Diseases COVID-19 Working Group, Abbott S, Kucharski AJ, Funk S. Estimating the overdispersion in COVID-19 transmission using outbreak sizes outside China. *Wellcome Open Res* 2020; **5**: 67. DOI:10.12688/wellcomeopenres.15842.1.

22 Scientific Advisory Group for Emergencies. SPI-M-O: Consensus statement on COVID-19, 1 July 2020. 2020. https://www.gov.uk/government/publications/spi-m-o-consensus-statement-on-covid-19-1-july-2020 (accessed July 31, 2020).

23 Davies NG, Kucharski AJ, Eggo RM, *et al.* Effects of non-pharmaceutical interventions on COVID-19 cases, deaths, and demand for hospital services in the UK: a modelling study. *Lancet Public Health* 2020; published online June. DOI:10.1016/S2468-2667(20)30133-X.

24 Zhang S, Diao M, Yu W, Pei L, Lin Z, Chen D. Estimation of the reproductive number of novel coronavirus (COVID-19) and the probable outbreak size on the Diamond Princess cruise ship: A data-driven analysis. *Int J Infect Dis* 2020; **93**: 201–4. DOI:10.1016/j.ijid.2020.02.033.

25 Lewer D, Menezes D, Aldridge RW. Hospital Readmissions Among People Experiencing Homelessness: A Cohort Study of Linked Hospitalisation and Mortality Data in England for 3,222 Homeless Inpatients. *Lancet Prepr* 2019. https://papers.ssrn.com/sol3/papers.cfm?abstract_id=3475583 (accessed April 1, 2020).

26 Benavides AD, Nukpezah JA. How Local Governments Are Caring for the Homeless During the COVID-19 Pandemic. *Am Rev Public Adm* 2020; **50**. DOI:10.1177/0275074020942062.

27 Conway B, Truong D, Wuerth K. COVID-19 in homeless populations: unique challenges and opportunities. *Future Virol* 2020; **15**. DOI:10.2217/fvl-2020-0156.

28 Van Slyke A. Homelessness during COVID-19: Understanding and Preventing Risk of Virus Spread in this Vulnerable Population. Lerner Center for Public Health Promotion, 2020 https://lernercenter.syr.edu/2020/06/22/ib-32/ (accessed Aug 3, 2020).

29 Henwood BF, Redline B, Lahey J. Surveying Tenants of Permanent Supportive Housing in Skid Row about COVID-19. Public and Global Health, 2020 DOI:10.1101/2020.04.17.20070052.

30 Gowda GS, Chithra NK, Moirangthem S, Kumar CN, Math SB. Homeless persons with mental illness and COVID pandemic: Collective efforts from India. *Asian J Psychiatry* 2020; **54**: 102268. DOI:10.1016/j.ajp.2020.102268.

31 Baggett TP, Keyes H, Sporn N, Gaeta JM. COVID-19 outbreak at a large homeless shelter in Boston: Implications for universal testing. *medRxiv* 2020. DOI:10.1101/2020.04.12.20059618.

32 Ly TDA, Hoang VT, Goumballa N, *et al.* Screening of SARS-CoV-2 among homeless people, asylum seekers and other people living in precarious conditions in Marseille, France, March April 2020. *MedRxiv* 2020; published online May 11. DOI:10.1101/2020.05.05.20091934v2.

33 Bartels SJ, Baggett TP, Freudenreich O, Bird BL. COVID-19 Emergency Reforms in Massachusetts to Support Behavioral Health Care and Reduce Mortality of People With Serious Mental Illness. *Psychiatr Serv* 2020; published online June 3. DOI:10.1176/appi.ps.202000244.

34 Bodkin C, Mokashi V, Beal K, *et al.* Pandemic Planning in Homeless Shelters: A pilot study of a COVID-19 testing and support program to mitigate the risk of COVID-19 outbreaks in congregate settings. *Clin Infect Dis* 2020; published online June 8. DOI:10.1093/cid/ciaa743.

35 Tobolowsky FA, Gonzales E, Self JL, *et al.* COVID-19 Outbreak Among Three Affiliated Homeless Service Sites — King County, Washington, 2020. *Morb Mortal Wkly Rep* 2020; **69**. DOI:10.15585/mmwr.mm6917e2.

36 Baggett TP, Keyes H, Sporn N, Gaeta JM. Prevalence of SARS-CoV-2 Infection in Residents of a Large Homeless Shelter in Boston. *JAMA* 2020; **323**. DOI:10.1001/jama.2020.6887.

37 Mosites E, Parker EM, Clarke KEN, *et al.* Assessment of SARS-CoV-2 Infection Prevalence in Homeless Shelters — Four U.S. Cities, March 27–April 15, 2020. *MMWR Morb Mortal Wkly Rep* 2020; **69**. DOI:10.15585/mmwr.mm6917e1.

38 Miyawaki A, Hasegawa K, Tsugawa Y. Lessons from Influenza Outbreaks for Potential Impact of COVID-19 Outbreak on Hospitalizations, Ventilator Use, and Mortality Among Homeless Persons in New York State. *J Gen Intern Med* 2020; published online June 4. DOI:10.1007/s11606-020-05876-1.

39 Baggett TP, Lewis E, Gaeta JM. Epidemiology of COVID-19 among people experiencing homelessness: Early evidence from Boston. 2020. https://deepblue.lib.umich.edu/handle/2027.42/154734 (accessed Aug 3, 2020).
